# Epicardial Adipose Tissue in Diabetic Heart Disease: Impact on Cardiac Function and Modulation Strategies, a Comprehensive Review

**DOI:** 10.3390/medicina62071402

**Published:** 2026-07-20

**Authors:** Ana Đuzel Čokljat, Petra Grubić Rotkvić, Zdravko Babić, Ivana Huljev Šipoš, Marijo Bekić, Marina Njire Bratičević, Luka Rotkvić, Maja Cigrovski Berković

**Affiliations:** 1 Department of Internal Medicine, General Hospital Dubrovnik, 20000 Dubrovnik, Croatia; 2 Department of Cardiovascular Diseases, University Hospital Centre Zagreb, 10000 Zagreb, Croatia; 3 Department of Cardiovascular Diseases, University Hospital Centre “Sestre Milosrdnice”, 10000 Zagreb, Croatia; 4 Department of Pulmonology, University Hospital Dubrava, 10000 Zagreb, Croatia; 5 Department of Orthopedics and Traumatology, General Hospital Dubrovnik, 20000 Dubrovnik, Croatia; 6 Medical Biochemistry Laboratory, General Hospital Dubrovnik, 20000 Dubrovnik, Croatia; 7 Clinic for Cardiovascular Medicine Magdalena, 49217 Krapinske Toplice, Croatia; 8 Faculty of Kinesiology, University of Zagreb, 10000 Zagreb, Croatia; maja.cigrovskiberkovic@gmail.com

**Keywords:** epicardial adipose tissue, diabetic heart disease, type 2 diabetes, heart failure, obesity, antidiabetic medications

## Abstract

Epicardial adipose tissue (EAT) is a distinct form of visceral adipose tissue that lies within the pericardium and directly adjacent to the myocardium. Individuals with type 2 diabetes mellitus (T2DM) exhibit excessive and metabolically active EAT, which contributes to the development of early diabetic myocardial disease, formerly referred to as diabetic cardiomyopathy. Recent studies have demonstrated that excess EAT is characterized by a proinflammatory profile that may adversely affect the underlying myocardium, leading to impaired diastolic and systolic function. In this review, we discuss the role of excessive EAT as a source of proinflammatory and profibrotic cytokines that influence adjacent ventricular and atrial myocardium through local tissue crosstalk. In addition to metabolic alterations, enlarged EAT induces hemodynamic changes that result in pericardial constraint and enhanced ventricular interdependence, both of which are hallmarks of diabetic pericardial disease. We further analyze the interplay among T2DM, inflammation, obesity, and increased EAT on the one hand, and myocardial dysfunction characterized by myocardial stiffness, elevated filling pressures, and diastolic and systolic dysfunction on the other. We emphasize that the distinct immunometabolic activity of perivascular adipose tissue may lead to a paradigm shift in the understanding of coronary artery disease, moving from a predominantly endoluminal to an exoluminal perspective. A wide range of dietary, lifestyle, and pharmacological interventions are available within this emerging diabeto-cardiometabolic continuum, each with a potential role; however, the timing of intervention is crucial. This review also explores the potential effects of antidiabetic and other pharmacological agents that modulate EAT thickness, volume, and/or activity, and discusses directions for future mechanistic and clinical research.

## 1. Introduction

### 1.1. EAT’s Distinctive Metabolic Profile

Epicardial adipose tissue (EAT) is a separate form of visceral adipose tissue (VAT) located between the myocardium and the visceral layer of the pericardium (epicardium). Since EAT lacks a fascial boundary from the underlying myocardium, these tissues share the same microcirculation. In addition to paracrine and vasocrine activity, EAT may function as an endocrine organ, which can increase inflammatory burden in affected individuals [1,2]. As opposed to subcutaneous adipose tissue (SAT), EAT contains thickened connective tissue septa with dense inflammatory cell infiltrates that extend to the fat lobules [3]. EAT is characterized by the presence of T lymphocytes, macrophages, and mast cells, differentiating it from SAT [3]. EAT exhibits a distinct gene expression profile that suggests its role as a local source of adipokines, which influence the myocardium through paracrine signaling [4]. The tissue’s distribution near cardiac structures, particularly with 75% located adjacent to the right ventricle, suggests that EAT may have significant effects on this region [5]. Variations in EAT transcriptome and proteome across different anatomical sites imply diverse impacts on cardiac health, potentially leading to various cardiovascular (CV) complications [4,6,7]. In type 2 diabetes mellitus (T2DM), EAT shows increased levels of advanced glycation end products (AGEs) and their receptors (RAGEs), which contribute to oxidative stress and amplify its atherogenic properties [8]. Notably, AGEs, rather than glucose, are shown to elevate interleukin (IL)-6 expression [9]. The transcriptome of EAT in T2DM patients differs significantly from that of SAT, highlighting heightened involvement of innate immune response and lipid metabolism pathways, primarily driven by the AGE-RAGE signaling mechanism [8]. In contrast, the gene profiles of SAT from T2DM and non-T2DM individuals are largely comparable, underscoring the pivotal role of inflammatory signaling in the pathophysiology of EAT transformation.

### 1.2. EAT’s Physiologic Role and Its Pathologic Transformation

**Figure 1:**
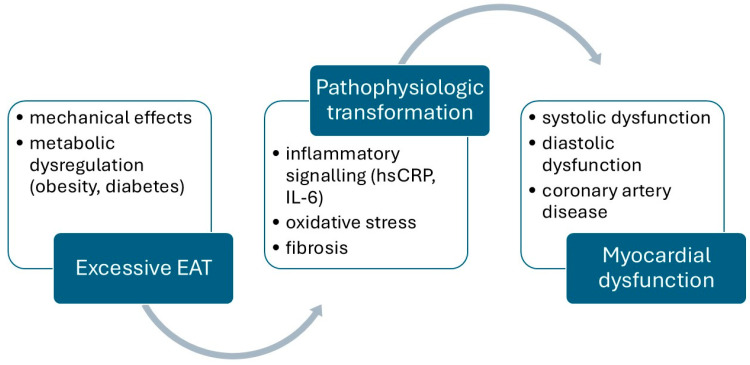
EAT’s pathologic transformation.

EAT is a major source of energy and free fatty acids (FFA) for the adjacent myocardium. During early development, it provides thermogenic function similar to brown adipose tissue (BAT). Prior imaging studies have shown that BAT is associated with optimal fat distribution and lower prevalence of T2DM independently of total or regional adiposity [10]. Later in life, EAT acts as a buffer supplying the myocardium with FFA but also preventing excess fatty acid influx [11]. However, under pathological conditions, EAT can affect the myocardium through excessive and abnormal lipid influx. The “cardio-lipotoxicity” of the EAT is complex and involves different pathways, such as increased inflammation, the infiltration of lipid intermediates, mitochondrial dysfunction, and oxidative stress, ultimately leading to cardiomyocyte dysfunction and coronary artery ischemia (Figure 1). These changes can contribute to the pathogenesis of various cardio-metabolic diseases including T2DM, atrial fibrillation (AF), coronary artery disease (CAD), and heart failure (HF), namely HF with preserved ejection fraction (HFpEF), which will be discussed later. Genetic, epigenetic, and environmental factors can initiate a transformation from a protective to a maladaptive form of EAT, promoting the proinflammatory and profibrotic phenotype. Epidemiological data have linked circulating levels of inflammatory cytokines, such as IL-6 and tumor necrosis factor (TNF)-α or their hepatic product, C-reactive protein, with long-term CV risk in apparently healthy populations as well as those with already established CAD [12,13,14]. In addition to a vascular origin, these inflammatory mediators may also derive from remote extravascular sources (i.e., endocrine), thus providing a mechanistic explanation for increased CV risk in certain populations, such as insulin-resistant individuals who manifest hypersecretion of cytokines from adipose tissue [15,16]. Since adipose tissue expresses TNF-α and IL-6, it may cause obesity-related insulin resistance.

Taken together, growing evidence suggests that EAT is not merely a passive fat depot, but a metabolically active and proinflammatory tissue that contributes to myocardial dysfunction through local inflammatory, profibrotic, and mechanical interactions, particularly in the setting of T2DM. Given its emerging role in the diabeto-cardiometabolic continuum and its potential as a therapeutic target, we conducted a comprehensive review of the current literature to better define the pathophysiological mechanisms and clinical implications of EAT.

### 1.3. Literature Search

Although this manuscript is not intended as a systematic review, a detailed protocol for the search strategy of this review was developed prospectively, specifying objectives, study selection criteria, outcomes, and statistical methods. To detect all available studies on the association between CV disease and EAT, a systematic search was conducted in the electronic databases (PubMed, Web of Science, and Scopus). The search string applied to PubMed was the following: (Epicardial Adipose Tissue OR EAT) AND (thickness OR volume) AND (Diabetes mellitus type 2 OR T2DM OR Obesity OR HFpEF OR heart failure OR AFib OR atrial fibrillation OR CAD OR coronary artery disease OR VT OR ventricular tachycardia). To detect all available studies on the association between cardiometabolic drug effects and EAT, a systematic search was conducted in the electronic databases (PubMed, Web of Science, and Scopus). The search string applied to PubMed was the following: (Epicardial Adipose Tissue OR EAT OR EAT modulation OR EAT therapy) AND (thickness OR volume) AND (Diabetes mellitus type 2 OR T2DM) AND (glucagon-like peptide 1 OR GLP-1 OR GLP1 OR GLP1-agonist OR GLP-1 RA OR sodium glucose cotransporter 2 OR sodium–glucose co-transporter-2 OR SGLT2 OR SGLT-2 OR SGLT2-Inhibitors OR metformin OR thiazolidinediones OR glitazones OR dipeptidyl peptidase-4 inhibitors OR DPP-4 inhibitors OR sulfonylureas OR glinides OR insulin OR statin OR colhicine OR PCSK9i OR RAAS inhibitors OR trimetazidine OR diet OR body weight OR physical activity OR hypoxia OR obstructive sleep apnea). The last search was performed in June 2026.

## 2. Excessive EAT-Related Disease

### 2.1. Obesity

Obesity is a growing epidemic worldwide and is a major risk factor for T2DM, metabolic dysfunction-associated steatotic liver disease (MASLD), and CV disease. These diseases are associated with accumulation of lipids in non-adipose tissues (i.e., ectopic lipids). Excessive lipid accumulation in cells other than adipocytes, so-called “ectopic” cells, can ultimately lead to apoptotic cell death. Ectopic lipid accumulation in cells and tissues is accompanied by activation of oxidative, endoplasmic reticulum (ER), and proinflammatory stress pathways, as well as accumulation of lipid intermediates such as diacylglycerol and ceramides [17,18,19,20]. This lipid overload can impact various cellular signaling pathways and functions that have been referred to as “lipotoxic” [21]. Moreover, all these changes lead to a vicious circle created by the promotion of systemic inflammation resulting in further EAT accumulation, which in turn promotes local inflammation [22]. Necrotic white adipose tissue, a hallmark of obesity, is a driving force of macrophage infiltration. Over 90% of adipocyte tissue macrophages have been shown to accumulate and surround apoptotic adipocytes, forming a crown-like structure that is associated with increased TNF-α and IL-6 secretion [23,24]. Adipose tissue-derived TNF-α promotes endothelial dysfunction by making endothelial cells more receptive to inflammatory cells, and also displaying a more proinflammatory secretome, epitomized by IL-6 secretion [25]. Adipocyte-derived TNF-α acts mainly in an autocrine fashion, impairing signaling via the insulin receptor and increasing lipolysis with the subsequent release of non-esterified fatty acids that contribute to insulin resistance in the peripheral tissues. In contrast, IL-6 accentuates systemic low-grade inflammation and hepatic production of C-reactive protein and inhibits lipoprotein lipase [26]. In addition to adipokines secreted by adipocytes, lipotoxicity-induced changes in protein secretion include hepatokines, myokines, cardiokines, and osteokines secreted by hepatocytes, skeletal muscle cells, cardiac cells, and osteoblasts, respectively [21]. Future investigations are needed to clarify whether there are unified responses in protein secretion in order to protect other tissues against further metabolic stress in case of lipotoxicity, or whether these changes in cytokine secretion in fact promote further metabolic deterioration.

### 2.2. T2DM

There is a strong correlation between obesity and insulin resistance. This is proven in both individuals with T2DM and without T2DM, and the risk of T2DM increases 11-fold as the body mass index (BMI) increases from 20 to 30 [27,28]. The pathogenesis of insulin resistance is directly associated with chronic low-grade inflammation during obesity, which, in turn, promotes gradual and sustained molecular profile changes in the adipose tissue [29]. Although insulin resistance accompanies all obese individuals, the degree of insulin resistance is variable, and the relations between obesity, T2DM, and insulin resistance are not completely elucidated. Obesity-related insulin resistance may be attributed to proinflammatory cytokines secreted by adipose tissue that make some individuals more insulin resistant than others. Obesity-promoted adipose tissue hyperplasia and hypertrophy are often associated with an increased secretion of adipose tissue-derived cytokines-adipokines. The first ‘adipokine’ described to be produced from adipose tissue was the aforementioned TNF-α, which is elevated in obesity and contributes to obesity-associated metabolic disease [30]. Clinical studies have shown that individuals with obesity have increased serum levels of TNF-α that decline with weight loss [31]. As an important step for a comprehensive understanding of T2DM development, the authors of a quantitative proteomics study proposed the crucial role of TNF-α overexpression [32]. This study provides insight into crosstalk between molecular mechanisms engaged in disrupting insulin signaling and other inflammatory pathways by TNF-α that causes dynamic changes in the proteome landscape of adipocytes chronically treated with TNF-α. Future investigations should elucidate potential therapeutic targets for insulin resistance and T2DM. The study results confirmed that the overexpression of cytokines directly correlated with macrophage recruitment, particularly macrophage migration inhibitory factor [32,33]. As a result, T2DM is a paradigm of chronic, mostly low-grade inflammatory metabolic disease. The related cardiac conditions, namely diabetic myocardial disorder and diabetic pericardial disorder, will be discussed later.

Throughout the researched literature, most individuals with T2DM were overweight/obese, as defined by a BMI ≥ 25 kg/m^2^. However, unlike EAT, normal weight is not a direct measure of adiposity based on BMI. EAT is a marker of visceral adiposity, which represents a risk factor for the development of T2DM and CV complications attributed to adiposity. The inability of BMI to reflect cardiometabolic risk is partly related to the fact that BMI alone is an insufficient biomarker of total body, and especially, central abdominal fat mass. Additionally, BMI does not account for the extreme variation in VAT distribution in the population.

Therefore, future investigations of EAT’s role in CV risk estimation and EAT’s mediation in disease progression should focus on anthropometric measures, such as waist circumference, waist-to-height ratio, and height-to-EAT ratio, when describing the population of interest. Furthermore, corresponding EAT reference values should be established in order to encompass gathered knowledge, and to identify different specific groups of patients in the trajectory of the EAT-BMI correlation, which could redefine obesity itself.

### 2.3. CAD

Experimental laboratory investigations, comprehensive characterization of human vascular lesions, as well as myriad studies have all provided abundant evidence that inflammation plays a central role in the development and progression of atherosclerosis [34]. The signaling that originates from the vessel lumen or at the endothelial surface plays a cardinal role in the development of atherosclerotic lesions. There is growing evidence that changes in the perivascular tissues could also alter vascular homeostasis, which was initially established for the coronary arteries of the T2DM-porcine model and confirmed by the T2DM rat model, where perivascular adipose dysfunction contributed to oxidative stress, inflammation, endothelial dysfunction, and vasoconstriction [9,35]. EAT could contribute to CAD by macrophage activation, innate inflammatory response, oxidative stress, and plaque destabilization [3]. Mazurek et al. demonstrated significantly higher expression of chemokine (monocyte chemotactic protein [MCP]-1) and inflammatory cytokines (IL-6, IL-1β, and TNF-α) in EAT than in SAT in patients with established CAD [3]. In this study, EAT was collected near the proximal right coronary artery, and SAT was collected from the leg near the vein harvesting. Since this study showed significantly higher levels of investigated inflammatory mediators in EAT versus SAT, these findings illustrate regional proinflammatory properties of EAT. Conversely, plasma inflammatory biomarkers may not adequately reflect local tissue inflammation, so further studies are needed to establish novel non-invasively obtained (plasma) biomarkers. Since EAT inflammation was independent of obesity and T2DM in individuals with CAD in this study, it is possible that inflammation itself plays a crucial role by tissue crosstalk [3]. Perivascular adipose tissue could shift the endoluminal paradigm to the extraluminal, highlighting the need for a better understanding of comprehensive vascular biology. A recent cohort study results show that EAT volume was significantly associated with stress-induced myocardial ischemia in asymptomatic individuals with T2DM, and this association remained significant after controlling for gender, T2DM duration, peripheral macrovascular disease, and coronary artery calcium (CAC) score [36]. An Italian study involving individuals with systolic HF demonstrated a highly significant correlation between EAT thickness and the degree of cardiac sympathetic denervation [37,38]. In this context, it would be of particular interest to further investigate the potential relationship between EAT and silent ischemia in individuals with T2DM. Multiple pathways contribute to accelerated coronary atherosclerosis in T2DM, including increased oxidative stress and inflammatory burden. Emerging evidence indicates an active interplay between dysfunctional fat cells, visceral adiposity and T2DM-related coronary atherosclerosis [39]. However, the impact of T2DM on the EAT transcriptome in patients with CAD has not been sufficiently well elucidated so far. Firstly, the number of patients in former studies was relatively small (EAT samples were usually taken during CABG surgery), and heterogeneous due to co-existing chronic illnesses. Secondly, most of the studies used the microarray technique, whereas whole transcriptome sequencing analysis (RNA-seq) of EAT was seldom done. RNA-seq has several advantages over microarray, such as encompassing the whole transcriptome, allowing for the analysis of novel transcripts, splice junctions and noncoding RNAs. A study exploring the rat model of myocardial infarction proposed the EAT/microRNA (miRNA) axis as a new pathological mechanism involved in adverse myocardial remodeling, manifesting pathologically as myocardial hypertrophy and fibrosis [40]. A recent study of patients undergoing cardiac surgery confirmed that human EAT-derived miRNAs exert paracrine effects on the human heart, which improves the myocardial redox state and is associated with improved clinical outcomes [41]. However, the exact time and type of (non)pharmacological intervention in order to improve myocardial metabolism remains to be established.

### 2.4. AF

The hallmark of AF is atrial structural remodeling, primarily fibrosis, which results from excessive extracellular matrix (ECM) production by fibroblasts. Among multiple mechanistic possibilities, the metabolically active EAT has emerged as a potentially important factor involved in atrial remodeling [42]. A study in patients undergoing aorto-coronary bypass surgery showed that EAT infiltrates adjacent atrial myocardium, affects atrial conduction, and may trigger AF [43]. EAT secretome induces ECM gene expression in atrial fibroblasts and contains abundant myeloperoxidase (MPO), which is the most increased protein in the EAT secretome of patients with AF [44]. This study emphasizes an important association between EAT neutrophil activity and the fibrotic substrate of AF. Atrial EAT is a neutrophil-rich tissue that secretes numerous profibrotic molecules, including increased MPO in AF patients; hence, MPO may serve as a marker of structural remodeling in AF [45]. Given that EAT MPO was already increased in patients who had developed AF during the study follow-up, compared to patients who did not develop AF during the follow-up, atrial EAT may play a role in the formation of the arrhythmogenic substrate. Therefore, both neutrophils and MPO are promising targets for future research regarding arrhythmia prevention or therapy. The volume of EAT has been identified as an independent risk factor for the onset, severity, and recurrence of AF [45]. Obesity is associated with greater atrial conduction abnormalities and an increasingly remodeled atrial substrate that predisposes to AF [46]. However, whether obesity alone is responsible for all the electropathological abnormalities related to AF and to what extent this effect is mediated by EAT remains to be further investigated.

### 2.5. VT

Increased EAT thickness, as measured by ultrasound, is associated with fragmented QRS waves and prolonged QRS wave duration, suggesting its potential role in slowing ventricular conduction and promoting re-entry [47]. Increased EAT volume, as measured by computed tomography (CT), is associated with frequent premature ventricular contractions [48]. The connection between EAT and arrhythmias is proposed to occur through several mechanisms: (1) increased EAT may act as a physical barrier, slowing activation and altering cardiac electrophysiology, (2) EAT deposition may trigger lipid overload and the production of reactive oxygen species, contributing to both early and delayed afterdepolarizations, and (3) adipokines released by EAT may promote fibrosis and electrical remodeling [49,50]. A higher EAT, as measured by cardiac magnetic resonance (CMR), may be associated with ventricular tachycardia (VT) recurrence among individuals undergoing VT ablation [51]. Future research should focus on exploring the mechanisms by which EAT influences VT incidence, identifying biomarkers for EAT-related VT risk, and evaluating the efficacy of pharmacological interventions targeting EAT in reducing VT risk. Additionally, the association between EAT infiltration into the myocardium and sudden cardiac death has yet to be fully clarified.

## 3. Diabetic Heart Disease

Previously known as diabetic cardiomyopathy, diabetic heart disease implies structural and functional changes in heart muscle that develop and progress independently of concomitant macrovascular and microvascular diabetic complications. Histologically, this disorder is characterized by the development of myocardial fibrosis, cardiomyocyte hypertrophy, and apoptosis. Pathophysiologically, this entity is characterized by a myriad of impaired contractility/relaxation, interstitial fibrosis, dysregulated autophagy, microvascular dysfunction and impaired neuromodulation [52].

### 3.1. Diabetic Myocardial Disorder

Diabetic myocardial disorder is defined as systolic and/or diastolic myocardial dysfunction in the presence of diabetes [53]. Diabetes is seldom solely responsible for myocardial dysfunction, but usually acts in association with comorbidities, such as arterial hypertension, obesity, chronic kidney disease, CAD, and/or dyslipidemia, causing additive myocardial dysfunction. Several potential mechanisms contributing to the development of HF in T2DM include renin–angiotensin–aldosterone system (RAAS) activation, mitochondrial dysfunction, oxidative stress, inflammation, changes in intracellular calcium homeostasis, increased formation of AGEs, and myocardial energy substrate alterations including increased FFA utilization, decreased glucose utilization, and increased oxygen consumption, resulting in decreased cardiac efficiency [54,55,56]. Despite meticulous control of metabolic risk factors in T2DM (e.g., elevated HbA1c, BMI, hypertension, dyslipidemia), diastolic dysfunction remains in the absence of left ventricular remodeling or systolic impairment [57]. The optimal approach to timely recognize and diagnose diabetic myocardial disorder and to manage accordingly remains to be established.

### 3.2. Diabetic Pericardial Disorder

Previous systematic review and meta-analysis showed a significantly larger EAT in individuals with T2DM than non-T2DM individuals [58]. Whether EAT enlargement itself promotes maladaptive cardiac remodeling, or whether excessive EAT develops unfavorable metabolic changes in order to promote myocardial remodeling, is a matter of further investigation. A recently proposed syntagma “diabetic pericardial disorder” includes both unfavorable metabolic and hemodynamic effects of EAT with resulting primarily diastolic left ventricular dysfunction, but also impaired systolic function, both of which contribute to a higher risk of incident HF in patients with T2DM [59]. Different mechanisms of EAT involvement in the HFpEF syndrome have been investigated in a study by van Woerden et al. [60]. Two essential concepts of EAT involvement in HFpEF have been proposed. The first is related to infiltration of EAT into the adjacent myocardium, which disrupts myocardial architecture, stimulates myocardial thickening, and promotes diastolic dysfunction [43]. These changes are linked with the release of proteins that disrupt intermyocyte adhesion, modulate cellular metabolism, and increase inflammation. The second concept of EAT involvement with HFpEF comprises a mechanical effect of thickened EAT similar to that of constrictive pericarditis. Specifically, if a pericardial dilatation is non-congruent with EAT expansion, the adjacent myocardium cannot further dilate in cases of demanding hemodynamic conditions due to limited pericardial pliability. Increased EAT volume, surrounded by a relatively stiff pericardial sac, constrains the myocardium, resulting in diastolic dysfunction and elevated left ventricular filling pressure. In this context, invasive hemodynamic testing in individuals with HFpEF and thickened EAT confirmed an increased left ventricular eccentricity index, indicating that increased left and right ventricular coupling is directly related to excessive EAT thickness due to pericardial constraint [61,62]. The clinical implications demand prompt focus on therapeutic weight loss, thereby reducing circulating plasma volume and unloading the restricted and constrained heart. Moreover, weight loss is associated with a reduction in EAT in obesity-related HFpEF [63]. Notably, aforementioned studies involving invasive hemodynamic testing were performed with individuals in the supine position, which could exaggerate the mechanical effects of EAT [61,62]. In the supine position, increased venous return and elevated cardiac filling pressures can amplify the constrictive effects exerted by epicardial and pericardial structures. On the other hand, individuals with excess EAT might be better responders to novel therapies targeting weight loss. Since HFpEF is a heterogeneous syndrome, phenotyping individuals with HFpEF into several pathophysiologically homogenous groups may enable better targeting of treatment.

The prevention of clinical HF remains a major challenge in the treatment of patients with T2DM. Among this population, patients with asymptomatic cardiac structural/functional abnormalities represent a group with the greatest potential to benefit from early treatment. Considering the distinctive property of EAT having a shared microcirculation with the underlying myocardium, excessive EAT may serve as a marker of diabetic pericardial disorder with corresponding metabolic and hemodynamic implications.

## 4. EAT’s Impact on Myocardial Function

Diastolic dysfunction often precedes systolic dysfunction because diastole is an active, highly energy-dependent process, while systole is a cruder, muscle-driven contraction that can rely on compensatory mechanisms for a longer period of time. However, few studies have evaluated the effects on systolic and diastolic function separately. Instead, they evaluated early signs of heart failure, namely diastolic dysfunction. In study by Koepp et al., individuals with excess EAT, defined as EAT thickness ≥9 mm measured on the free wall of the right ventricle (RV) by echocardiography in the parasternal long-axis view, showed similar left ventricle (LV) dimensions, volumes, mass, left ventricular ejection fraction (LVEF), global longitudinal strain (GLS), and diastolic chamber stiffness, compared to individuals without excess EAT [62]. Conversely, estimated LV filling pressures assessed by the E/e′ ratio tended to be greater in patients with excess EAT. Although the study focused on the obese phenotype of HFpEF defined by BMI 30 kg/m^2^ and found that mean BMI was higher in individuals with excess EAT compared with patients without excess EAT, these results are potentially confounded by discrimination between excess EAT, elevated BMI, larger general adiposity, and their permutation. Another study found that, compared to both non-obese HFpEF and controls, individuals with obese HFpEF (defined as BMI ≥ 35kg/m^2^) displayed increased EAT thickness and greater total epicardial heart volume [64]. Epicardial fat thickness was 20 and 50% higher in obese HFpEF as compared to non-obese HFpEF and controls, respectively [64].

### 4.1. EAT’s Impact Primarily on Systolic Heart Disease

In a study of asymptomatic T2DM individuals with matched controls, T2DM was an independent predictor for impaired GLS, systolic strain rate, and early diastolic strain rate on multiple linear regression analysis. In this early diabetic myocardial disorder, LV longitudinal systolic and diastolic function were impaired, but the circumferential and radial functions were preserved [65]. These subclinical abnormalities in contractility are widely considered a precursor to the onset of clinical HF in T2DM. The mechanistic pathways described for EAT’s influence on impaired myocardial mechanics, epitomized by strain parameters, before changes in EF become apparent, are emerging. In a longitudinal study of individuals without HF, CMR-measured EAT thickness over the RV free wall was correlated with GLS impairment [66]. Furthermore, thickened EAT was associated with a higher risk of incident HF, and NT-proBNP and GLS had a mediating effect for the association between EAT thickness and HF risk. Another study of 170 individuals with suspected CAD showed that, besides being a predictor of CAD severity, CT-measured EAT volume was significantly positively correlated with GLS [67]. In this study, EAT volume was also an independent predictor of LV diastolic dysfunction. A recent study of 68 individuals with dyspnea and signs of diastolic dysfunction confirmed an association between excess EAT volume by CMR and impaired atrial strain at rest and during exercise stress, and impaired ventricular strain during exercise stress [68]. In this study, regionally increased EAT was independently associated with functional impairment of the adjacent chambers. Moreover, since individuals with higher total EAT volume had impaired biventricular diastolic function during exercise stress only, while atrial strain was reduced already at rest, it could be regarded as an early sign of subclinical decompensation. Atrial muscular strength is weaker with less capacity to compensate for additional external pressure load induced by excessive EAT. Finally, atrial functional impairment precedes ventricular decompensation, which emphasizes the role of the atrium in diastolic dysfunction, and later in systolic dysfunction.

### 4.2. EAT’s Impact Primarily on Diastolic Heart Disease

**Figure 2:**
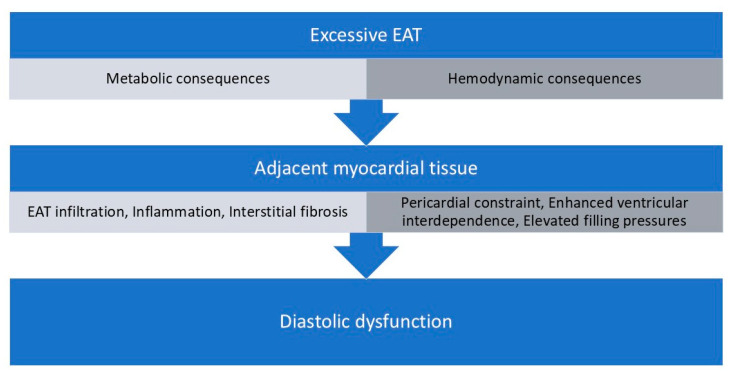
Diastolic dysfunction and EAT.

This risk for diastolic dysfunction and negative impact on LV geometry due to increased EAT thickness was found to be present in early adolescence, but also in adults without CAD [69,70]. Notably, there was no association between EAT thickness and LV ejection fraction. Individuals with normal systolic function and excess EAT volume had a reduced left atrial volume index (LAVI), lower E/e′ and E/A (*p* = 0.01), reduced e′ lateral and e′ septal, compared to individuals with lower EAT [71]. As late-diastolic a′ by tissue Doppler echocardiography reduces, reflecting further deterioration of LV compliance, while LAVI progressively increases, LAVI/a′ is a potential additive tool for detecting raised LV end-diastolic pressure [72]. In a study of individuals with chronic coronary syndrome and preserved LVEF, the authors showed that CT-measured EAT volume index (EAT volume divided by body surface area) correlated significantly with LAVI/a′ [73]. This study demonstrated that EAT volume index, and not CAD, was a robust predictor of LV diastolic dysfunction. A meta-analysis of 19 studies showed that EAT volume quantified by CT was associated with LA dilation, LV hypertrophy, and diastolic dysfunction defined by a higher E/e′ ratio, lower E′ velocity, and E/A ratio, independently of BMI [74]. Patients with diastolic dysfunction and increased EAT show more pronounced signs of diastolic dysfunction and adverse structural remodeling, in particular in the atria [68]. Echo-measured EAT thickness is significantly associated with LV diastolic dysfunction in T2DM patients in comparison to individuals without T2DM, indicating a more intimate relation to the myocardium associated with functional myocardial measures in T2DM [75]. Beyond myocardial stiffening, the important role in diastolic dysfunction is attributed to pericardial constraint. Since EAT is located directly adjacent to the myocardium, the presence of excess EAT, which is competing for space in a poorly pliable pericardial sac, might result in impaired diastolic function in cases of hemodynamic demand. The resulting elevated filling pressures and enhanced ventricular interdependence are a common finding in individuals with HFpEF who typically have abundant EAT [64]. Since excessive EAT is also attributed to T2DM, further studies should focus on early hemodynamic changes to enlighten early diabetic pericardial disorder in this group of patients. Figure 2 represents the impact of EAT on diastolic dysfunction.

## 5. Non-Pharmacological Modulation of EAT

**Figure 3:**
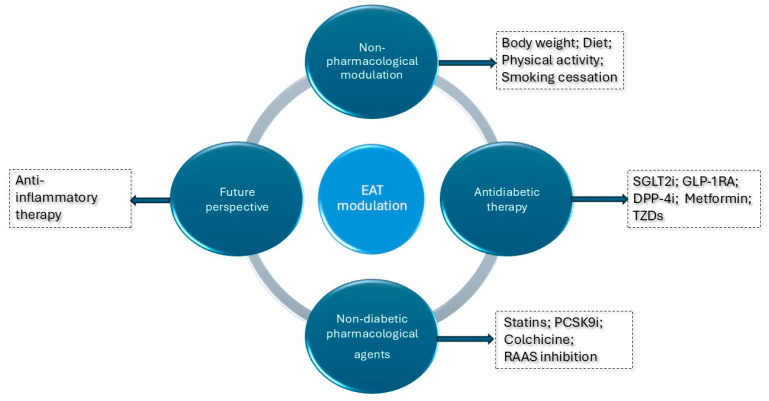
EAT modulation.

The phenotype of EAT is dynamic and can be influenced by a range of environmental and lifestyle-related factors. Several external determinants contribute to increases in EAT volume and thickness, often accompanied by enhanced inflammatory signaling. Modifying these factors may therefore represent a relevant strategy for mitigating the adverse CV effects associated with EAT, although it remains uncertain whether such interventions exert direct effects on EAT or act indirectly through overall improvement in cardiometabolic risk. Notably, most studies investigating non-pharmacological approaches have not been conducted exclusively in populations with diabetes mellitus (DM) and primarily rely on imaging-based assessments of EAT, with limited mechanistic exploration. Key targets for lifestyle-based modulation are outlined below, together with Figure 3 comprising the non-pharmacological and pharmacological strategies of EAT modulation.

### 5.1. Body Weight Control

As noted earlier, EAT represents a metabolically active visceral fat whose behavior cannot be fully understood through global measures of obesity alone. Although obesity is commonly defined using anthropometric indices such as BMI, this approach does not capture the biological heterogeneity of adipose tissue distribution. In contrast, adiposity reflects both the quantity and functional characteristics of individual fat depots. Among these, VAT is particularly relevant due to its high lipolytic activity and pro-inflammatory secretory profile, which contribute to systemic insulin resistance and cardiometabolic dysfunction. EAT shares these properties of visceral fat but differs functionally due to its anatomical proximity to the myocardium and coronary arteries, enabling direct local signaling effects on cardiac tissue, acting locally as a transducer of systemic inflammation [59].

Longitudinal imaging data indicate that EAT is not a fixed structure but responds to changes in systemic adiposity. Shifts in body weight, waist circumference, and BMI have been shown to be accompanied by corresponding changes in epicardial fat volume, supporting the concept that EAT appears to reflect systemic adiposity and metabolic status rather than acting independently [76]. More definitive evidence arises from interventional settings. Marked weight reduction, particularly following bariatric surgery, consistently leads to a decline in EAT alongside reverse cardiac remodeling and improvement in metabolic parameters. These findings collectively support the reversibility of epicardial fat under conditions of sustained negative energy balance [77,78,79,80]. Long-term observational evidence further suggests that reductions in visceral fat compartments, including EAT, are associated with sustained metabolic benefit over extended follow-up periods, underscoring its relevance as a modifiable cardiometabolic target [81]. Importantly, EAT does not appear to respond uniformly across all intervention types or time scales. In contrast to VAT and SAT, short-term lifestyle interventions may induce marked changes in peripheral fat depots without measurable modification of epicardial fat, suggesting a slower turnover rate and a degree of metabolic inertia specific to this depot [82].

From a clinical perspective, these observations are particularly relevant in DM, where dysfunctional adipose tissue expansion contributes to myocardial remodeling, impaired metabolic signaling, and progressive CV injury. Consequently, reduction in EAT through weight control may represent a mechanistic link between lifestyle-induced adiposity reduction and improvement in myocardial structure and function in patients with elevated cardiometabolic risk.

### 5.2. Diet

Dietary patterns represent an important modifiable component in the regulation of cardiometabolic risk, particularly in conditions such as obesity, DM, hypertension, and dyslipidemia, all of which are closely linked to alterations in EAT [83]. Rather than acting solely through caloric restriction, dietary composition influences systemic inflammation, insulin sensitivity, and visceral fat distribution, thereby indirectly modulating EAT.

Current CV prevention guidelines advocate adherence to a Mediterranean dietary pattern, which is characterized by high intake of plant-based foods, olive oil, and fish, and low consumption of processed products. This dietary pattern has been consistently associated with reduced visceral adiposity and improved metabolic profile, although specific data on EAT remain relatively limited [84]. Emerging evidence suggests that dietary quality may also influence epicardial fat directly. In a substudy of patients with AF undergoing ablation, higher adherence to the Mediterranean diet was associated with lower EAT volume, supporting a link between dietary pattern and this specific visceral fat depot [85].

Beyond dietary patterns, supplements, specific bioactive compounds (bioflavonoids) and nutraceutical approaches may contribute to metabolic improvement, particularly in prediabetes and diabetes. Although their direct effects on EAT remain under investigation, their beneficial influence on glycemic control and systemic inflammation suggests a plausible indirect role in modulating epicardial fat accumulation [86]. Vitamin D has been investigated in relation to adipose tissue distribution and cardiometabolic risk, with some studies showing reductions in visceral fat and improvements in insulin resistance, glycemic control, and selected lipid profiles with vitamin D supplementation. However, interventional data specifically addressing EAT are scarce and inconsistent, and therefore vitamin D supplementation should currently be considered a potentially relevant but not yet established strategy for EAT modulation [87].

Overall, dietary interventions may influence EAT through systemic improvement in metabolic and inflammatory status, which in turn reshapes visceral fat distribution.

### 5.3. Physical Activity

Physical activity is another key determinant of cardiometabolic health, with growing evidence supporting its role in the regulation of cardiac fat depots, including EAT. Most available data derive from heterogeneous populations rather than individuals with diabetes alone but consistently suggest that structured exercise interventions can modify epicardial fat accumulation [88,89,90,91,92]. Both aerobic and resistance training modalities appear capable of reducing EAT, suggesting that overall energy expenditure and metabolic activation rather than exercise type alone may be the key determinant. This is supported by short-term intervention data showing that different exercise strategies, including high-intensity interval training and moderate continuous training, lead to reductions in EAT volume regardless of exercise modality. Importantly, baseline EAT levels tend to be higher in metabolically impaired individuals, indicating that initial metabolic status may influence the magnitude of response [93].

In summary, physical activity may influence EAT through multiple pathways, including improved systemic lipid oxidation, enhanced insulin sensitivity, and reduced low-grade inflammation and oxidative stress, all of which contribute to reduced visceral fat accumulation.

### 5.4. Smoking Cessation

Smoking represents a well-established modifiable CV risk factor and remains a key target in both primary and secondary prevention of cardiometabolic disease. Beyond its systemic vascular effects, smoking has also been associated with increased EAT accumulation in clinical populations, including patients with metabolic syndrome, hypertension, and DM. Imaging studies using CT and echocardiography consistently report higher EAT volume or thickness in smokers compared with non-smokers, suggesting a relationship between tobacco exposure and visceral fat distribution [94,95,96]. This association is likely mediated through smoking-induced systemic inflammation, oxidative stress, and endothelial dysfunction, which collectively promote visceral fat dysregulation. Accordingly, smoking cessation may contribute not only to vascular protection but also to reduced visceral fat, including EAT accumulation.

### 5.5. Hypoxic Burden Control

Obstructive sleep apnea (OSA) represents another important condition linking lifestyle-related factors with cardiometabolic dysfunction and altered fat distribution. Obesity is a major risk factor for OSA, which is characterized by recurrent episodes of upper airway obstruction during sleep leading to intermittent hypoxia and reoxygenation cycles [97,98]. In this context, EAT has been identified as a metabolically active visceral fat depot that may represent a potential mechanistic link between OSA and CV disease [99]. Traditionally, OSA severity has been assessed using the apnea–hypopnea index (AHI); however, this measure does not fully capture the physiological burden of nocturnal hypoxia. More recently, hypoxic burden—defined as the cumulative magnitude and duration of oxygen desaturation—has emerged as a more robust predictor of CV risk and end-organ effects [100]. Intermittent hypoxia contributes to sympathetic activation, vascular dysfunction, and systemic metabolic disturbances, thereby promoting insulin resistance and cardiometabolic dysregulation [101]. These processes are closely linked to increased visceral adiposity, including expansion of EAT, as well as hepatic steatosis, hypertension, and impaired glucose metabolism [102]. Importantly, therapeutic strategies targeting nocturnal hypoxia appear capable of modifying cardiometabolic risk profiles. Continuous positive airway pressure (CPAP) therapy has been associated with reductions in EAT thickness and improvements in metabolic and vascular parameters, supporting a potential reversibility of hypoxia-driven adipose tissue dysfunction [103]. Nevertheless, optimal management likely requires a multifactorial approach combining weight reduction, regular physical activity, breathing interventions, and CPAP therapy.

Overall, these lifestyle-related factors—energy imbalance, dietary composition, physical inactivity, tobacco exposure, and intermittent hypoxia—converge on shared pathways involving systemic inflammation, oxidative stress, insulin resistance, and visceral fat dysfunction. Their convergence further supports a multifactorial model of EAT regulation. In conclusion, EAT emerges as a metabolically sensitive visceral fat that integrates environmental and cardiometabolic signaling, thereby representing a modifiable interface between lifestyle factors and cardiac function and not just a marker of CV disease.

## 6. Pharmacological EAT Modulation in Diabetes

### 6.1. Overview of Antidiabetic Therapy and EAT

Glycemic control in Type 1 Diabetes (T1DM) is primarily achieved through insulin therapy, whereas pharmacological management of T2DM is considerably more complex. This complexity arises from the increasing number of available agents with diverse mechanisms of action, whose effective clinical use requires a thorough understanding of their benefits as well as potential adverse effects. CV risk assessment plays a key role in selecting appropriate therapy for patients with T2DM, as drug classes differ substantially in both mechanisms and safety profiles. Consequently, novel antidiabetic agents undergo rigorous regulatory evaluation to ensure CV safety [104]. Effective management of DM is essential for preventing and delaying its complications. However, although EAT thickness and volume are markedly increased in DM, the mechanisms linking antidiabetic therapy to EAT modulation remain incompletely understood [83,105,106].

Patients with T2DM commonly present with abdominal obesity and increased visceral adiposity, including EAT. This phenotype is strongly associated with a higher risk of HFpEF. Moreover, antihyperglycemic therapies associated with weight gain have been linked to an increased risk of HF in clinical studies, potentially mediated by drug-induced adipogenesis [107,108,109]. In this context, EAT represents a pathophysiological link between metabolic dysfunction and HFpEF progression. Since EAT contributes to the pathophysiology of T2DM, it may represent a promising therapeutic target in CV risk reduction. However, no treatments that specifically target EAT metabolism and/or its reduction have been proven effective to date.

Insulin and non-insulin therapies used in the management of T2DM, with potential effect (both positive and negative) on EAT, are discussed in the following sections.

1. **Metformin**, a biguanide, has long been regarded as the first-line therapy for type 2 diabetes, in conjunction with dietary modification, weight reduction, and regular physical activity. Its primary mechanisms include suppression of hepatic glucose production through inhibition of gluconeogenesis and glycogenolysis, along with improved peripheral insulin sensitivity. As monotherapy, metformin exerts a potent glucose-lowering effect in a dose-dependent manner, with a low risk of hypoglycemia. Dose adjustment is required in accordance with renal function, and its use is contraindicated in patients with a glomerular filtration rate below 30 mL/min/1.73 m^2^, as well as in diabetic ketoacidosis, conditions associated with tissue hypoxia, and hepatic insufficiency [110,111]. Metformin has been shown to significantly reduce EAT thickness. Clinical studies report that 3 months of metformin monotherapy decreases EAT in both newly diagnosed T2DM patients and obese children with insulin resistance [112,113]. Beyond structural changes, metformin also influences EAT-related cardiac risk. Since EAT contributes to AF development, its reduction is clinically relevant. In parallel, metformin improves atrial electromechanical delay—a predictor of arrhythmias—suggesting a protective electrophysiological effect [113].

Mechanistically, metformin exerts pleiotropic effects-modulates EAT biology through anti-inflammatory and metabolic pathways. It activates AMPK and enhances PPARγ expression, leading to reduced oxidative stress, inhibition of NF-κB signaling, and increased adiponectin release. Additionally, metformin suppresses pro-inflammatory cytokines such as IL-6 and TNF-α. These changes promote anti-inflammatory and antioxidative effects, improving atrial remodeling [114,115,116,117,118,119]. Nevertheless, in the study of Iacobellis et al., metformin was not effective in reducing EAT thickness after 3–6 months of therapy in patients with T2DM, which highlights the need for further investigations [120].

2. **Thiazolidinediones** (glitazones; TZDs) are oral antidiabetic agents that enhance insulin sensitivity in the liver, adipose tissue, and skeletal muscle via activation of the nuclear transcription factor PPAR-γ. They also reduce hepatic glucose production and provide effective glycemic control without significantly increasing the risk of hypoglycemia. Although beneficial effects on CV outcomes have been observed in patients with macrovascular complications, their use may be associated with adverse effects such as congestive HF [121]. TZDs are associated with weight gain, raising concerns about their impact on EAT [122]. However, their effect appears to involve fat redistribution rather than simple accumulation. TZDs promote a shift from visceral fat (including EAT) to SAT [123]. Pioglitazone has been shown to reduce EAT thickness despite increasing overall body weight [124]. While it may increase pericardial fat volume, this change does not appear to impair myocardial function over short-term follow-up and may even improve diastolic function in patients with T2DM [125]. Additionally, pioglitazone exerts anti-inflammatory effects by suppressing inflammatory gene expression in EAT, particularly in patients with atherosclerosis [126]. Rosiglitazone demonstrates distinct metabolic effects, including rapid induction of EAT browning in experimental animal models characterized by increased expression of thermogenic and fatty acid oxidation genes. This shift enhances lipid turnover and contributes to its hypolipidemic action [127]. Despite this, its clinical use is limited by an increased risk of myocardial infarction [128]. Taken together, TZDs may have beneficial effects on EAT distribution, function, and inflammatory activity, although their net CV impact remains uncertain.
3. **Dipeptidyl peptidase-4** (DPP-4) inhibitors act by preventing the degradation of endogenous incretin hormones, including glucagon-like peptide-1 (GLP-1) and gastric inhibitory peptide (GIP). By increasing circulating levels of active incretins, they enhance glucose-dependent insulin secretion and suppress glucagon release, resulting in a very low risk of hypoglycemia. Their glucose-lowering efficacy is moderate, and they are generally well tolerated. Large CV outcome trials (CVOTs) have demonstrated no significant differences in CV risk compared with placebo for this class of agents, with the exception of an increased incidence of hospitalization for HF observed with saxagliptin (3.5% vs. 2.8%) [129,130]. Regarding EAT, sitagliptin has been shown to rapidly reduce its thickness, an effect that appears independent of changes in BMI and glycemia, suggesting additional mechanisms beyond glycemic and lipid-lowering actions [131]. Comparative data indicate that its effects on fat accumulation, cardiac function, and myocardial fatty acid metabolism are similar to those of empagliflozin [132]. However, experimental evidence on DPP-4 inhibition and cardiac remodeling remains inconsistent. Some studies report reduced myocardial fibrosis via decreased collagen type III production, while others show exacerbation of fibrosis, inflammation, and impaired ventricular function [133,134]. Although these findings do not directly address EAT, it is plausible that EAT may contribute to mediating the cardiac effects of DPP-4 inhibitors.

Overall, DPP-4 inhibitors may influence EAT and cardiac structure, but their CV effects are uncertain and potentially context-dependent, warranting further investigation.

4. **Glucagon-like peptide-1 receptor agonists** (GLP-1 RA; mimetics and analogs) bind to and activate the GLP-1 receptor, thereby enhancing glucose-dependent insulin secretion without causing significant hypoglycemia. They also modestly delay gastric emptying, which slows postprandial glucose absorption. These agents are typically administered subcutaneously, although oral formulations of semaglutide are available. Their resistance to degradation by the DPP-4 enzyme results in a prolonged half-life. GLP-1 RAs provide moderate to substantial reductions in HbA1c and are associated with weight loss. Meta-analyses of clinical trials indicate that they reduce the risk of major CV events in patients with T2DM and established atherosclerotic CV disease [104,135]. EAT has emerged as a relevant therapeutic target of GLP-1 RA, as it expresses GLP-1 receptors at higher levels than SAT, enabling direct drug effects independent of glycemic control [136]. The presence of GLP-1 receptors on cardiomyocytes further supports the pleiotropic CV actions of this drug class [137]. Clinical studies consistently demonstrate that GLP-1 RAs significantly reduce EAT thickness and volume, with reductions of approximately 20–30% after 3 months of therapy and up to 40% within 12–24 weeks, suggesting a class effect with dose dependency [120,138,139]. Meta-analytic data indicate that GLP-1 RAs exert a greater early impact on EAT reduction compared with other cardiometabolic agents, such as SGLT2 inhibitors and statins, although effects may become comparable with SGLT2 inhibitors over longer treatment durations. The magnitude of EAT reduction appears greater in younger individuals and those with higher BMI [140]. Mechanistically, GLP-1 receptor activation in EAT is associated with enhanced fatty acid β-oxidation and promotion of white-to-brown adipose tissue differentiation, supported by the upregulation of brown adipose tissue–related genes [141]. Additionally, GLP-1 RAs improve the metabolic and inflammatory profile of EAT by reducing triglyceride and low-density lipoprotein content, as well as systemic inflammatory and oxidative stress markers [142]. These changes are accompanied by improvements in vascular stiffness and cardiac function, indicating a link between EAT modulation and CV benefit [143,144]. Emerging dual incretin therapies, such as tirzepatide, may further expand this therapeutic paradigm due to combined GLP-1 and GIP receptor activity, although current evidence regarding their direct effects on EAT remains limited [83].

In conclusion, GLP-1 RAs exert significant quantitative and qualitative effects on EAT, which likely contribute to their cardioprotective properties beyond glucose lowering, highlighting EAT as a mediator in cardiometabolic risk reduction.

5. **Sodium–glucose cotransporters**: The kidneys play a crucial role in glucose homeostasis through reabsorption mediated by sodium–glucose cotransporters 1 and 2 (SGLT-1 and SGLT-2) [145]. Inhibitors of these transporters represent the newest class of oral antidiabetic agents and act by increasing urinary glucose excretion through lowering the renal threshold for glucose (in comparison to other medications that primarily work to utilize glucose). Large CVOTs have shown that SGLT2 inhibitors (SGLT2i) confer CV protection and reduce hospitalization for HF within weeks of initiation, regardless of baseline CV risk, and demonstrate a class effect. These findings suggest that mechanisms beyond glycemic control and atherogenesis may underlie their cardioprotective effects [146]. Emerging evidence suggests that SGLT-2i may exert cardioprotective effects partly through modulation of EAT. Experimental and clinical studies indicate that these agents reduce EAT, alter its metabolic activity, and attenuate its inflammatory profile, thereby potentially contributing to improved CV outcomes. Cinti et al. demonstrated that dapagliflozin induced a rapid reduction in epicardial fat thickness of approximately 19% within four weeks in patients with T2DM, with a more pronounced effect on EAT compared to other adipose depots, likely reflecting its higher metabolic activity [147]. However, these findings should be interpreted cautiously due to the small sample size. The work by Díaz-Rodríguez et al. showed that dapagliflozin increased glucose uptake in human EAT samples via glucose transporter 4 (GLUT4), suggesting a more complex and context-dependent metabolic effect [148]. Nevertheless, further mechanistic support comes from the study of Sato et al., who reported that dapagliflozin significantly decreased EAT at the 6-month follow-up compared with the conventionally treated patients with T2DM and CAD. Decrease in EAT volume was accompanied by reduction in TNF-α level and body weight [149]. Clinical data in non-diabetic patients with heart failure with reduced ejection fraction (HFrEF) further support the beneficial effects of SGLT-2 inhibition. Requena-Ibáñez et al. reported that empagliflozin significantly reduced epicardial fat volume and was associated with decreased interstitial myocardial fibrosis and improved aortic stiffness, with fibrosis assessed indirectly via extracellular volume fraction as a validated prognostic marker in HF. Comparative analyses of cardiometabolic therapies have produced mixed findings. Although in the previously mentioned study of Myasoedova et al. it was reported that GLP-1RA may be more effective than SGLT-2i in reducing EAT, Bao et al. suggested superiority of SGLT-2 inhibitors over GLP-1RA and exercise interventions in reducing EAT, highlighting ongoing uncertainty regarding the most effective pharmacological strategy [140,150]. Collectively, SGLT-2 inhibitors may improve CV outcomes by restoring the balance between pro- and anti-inflammatory adipokines, thereby influencing insulin resistance, atherosclerosis, coagulation, and fibrinolysis [151,152]. They have been shown to modulate adipokine secretion, including reductions in leptin and increases in adiponectin, as well as to improve adipocyte differentiation and reduce inflammatory cytokine release [153]. Reduced EAT volume and inflammation may contribute to decreased risk of HF, AF, and CAD, as well as prevention of adverse cardiac remodeling. Even so, limited mechanistic understanding of SGLT2 inhibitors’ mode of action underscores the need for further research in this area.
6. **Sulfonylureas** stimulate insulin secretion from pancreatic β-cells independently of plasma glucose levels, meaning they act in hyperglycemic, normoglycemic, and even hypoglycemic states. They are among the most potent oral agents for lowering blood glucose [154]. Some observational studies and meta-analyses have suggested a potential association between certain first-generation sulfonylureas, such as glibenclamide, and increased CV mortality. Still, this finding has not been consistently confirmed in randomized controlled trials [155].

To the best of our knowledge, no studies have directly assessed their effects on EAT.

7. **Glinides** are short-acting insulin secretagogues that, like sulfonylureas, stimulate insulin release from pancreatic β-cells. Their rapid onset and short duration of action necessitate administration immediately before meals, often requiring multiple daily doses. The risk of hypoglycemia is somewhat lower compared to sulfonylureas [156]. No data are available regarding their effects on EAT.
8. **Insulin** is an endocrine peptide hormone that interacts with plasma membrane-bound receptors on target cells, thereby coordinating a systemic anabolic response to nutrient availability [157]. It stimulates lipogenesis and lipid storage in adipose tissue [158]. Insulin therapy has been associated with an increased risk of HF, potentially mediated by EAT expansion and antinatriuretic effects [159,160,161,162]. Although data regarding EAT and insulin are limited, in a pilot study by Elisha et al., it has been shown that treatment with insulin analogs (detemir and glargine) among insulin-naïve uncontrolled patients with T2DM led to a reduction in EAT thickness, surprisingly. Furthermore, more pronounced EAT thickness reduction was observed with detemir [163].

### 6.2. Non-Diabetic Pharmacological Agents

Several non-diabetic pharmacological agents, including statins, proprotein convertase subtilisin/kexin type 9 (PCSK9) inhibitors, trimetazidine, mineralocorticoid receptor antagonist (MRA), angiotensin-converting enzyme (ACE) inhibitors, angiotensin receptor blockers (ARB), neprilysin inhibitors (ARNI—Angiotensin Receptor—Neprilysin Inhibitor), and colchicine, have also been associated with modulation of EAT and cardiometabolic risk [22,140].

1. **Statins**. Growing evidence indicates that statins exert CV benefits beyond low-density lipoprotein (LDL) reduction, partly through modulation of EAT. High-intensity statin therapy has been associated with reductions in EAT volume and inflammatory activity, effects that appear to occur independently of lipid lowering. By attenuating the pro-inflammatory profile of EAT and perivascular adipose tissue, statins may contribute to decreased systemic inflammation and reduced progression of coronary atherosclerosis [164,165,166,167,168,169,170,171,172]. Experimental and clinical studies further suggest that these pleiotropic effects may improve myocardial structure and function. Statin therapy has been linked to attenuation of left ventricular diastolic dysfunction (LVDD), myocardial fibrosis, and microvascular impairment, particularly in obesity, dyslipidemia, and T2DM. Improvements in diastolic function have been observed with atorvastatin, pravastatin, simvastatin and rosuvastatin, while intensive statin regimens appear more effective than moderate-intensity therapy. Animal studies similarly demonstrate prevention or reversal of diastolic dysfunction following statin treatment [173,174,175,176,177,178,179,180,181,182,183]. In light of their pleiotropic action, observational clinical studies additionally suggest that statin use may reduce mortality risk in patients with HFpEF, whereas comparable benefits have not been consistently observed in HFrEF [184,185,186,187,188,189]. Recent meta-analytic evidence reinforces the concept that statins can significantly reduce EAT, although the magnitude of this effect appears modest. Current evidence indicates that the reduction in EAT achieved with statin therapy may be dose-dependent and partially attributable to anti-inflammatory mechanisms, including suppression of inflammatory cytokine secretion and modulation of peroxisome proliferator-activated receptor (PPAR) pathways. These findings support the emerging paradigm that atherosclerosis involves not only the vascular wall but also surrounding perivascular adipose tissues [190,191,192,193].

Nevertheless, current evidence remains limited by heterogeneity in imaging methods, study design, and follow-up duration. Further randomized controlled trials are therefore required to clarify whether reductions in EAT directly mediate improvements in cardiac function and to determine the influence of statin intensity on these effects [194].

2. **PCSK9i**. Recent evidence demonstrating the expression of PCSK9 within EAT suggests that PCSK9 may constitute an important component of the EAT secretome. Moreover, EAT inflammation appears to be associated with increased local PCSK9 expression independently of circulating PCSK9 concentrations. These findings support the hypothesis that PCSK9 may contribute to the local inflammatory and metabolic activity of EAT, but further studies are required to determine whether targeted reduction in EAT inflammation or local PCSK9 expression may translate into improvements in EAT metabolism and reductions in CV risk [195]. In this context, PCSK9i, a novel class of lipid-lowering agents, have emerged as potential modulators of EAT expansion and dysfunction. Rivas-Gálvez et al. reported that treatment with the PCSK9 inhibitors evolocumab and alirocumab was associated with significant reductions in EAT volume after six months of therapy. These observations suggest that PCSK9 inhibition may exert pleiotropic cardiometabolic effects beyond low-density lipoprotein cholesterol reduction, potentially influencing adipose tissue remodeling and inflammatory pathways [196]. However, current evidence remains limited, particularly regarding the impact of PCSK9 inhibitors on cardiac diastolic function, warranting further investigation in larger prospective studies.
3. **Trimetazidine** (TMZ). Increased EAT thickness correlates with elevated concentrations of visfatin, an adipocytokine linked to obesity, insulin resistance, and CV disease. In addition to systemic secretion from adipocytes, visfatin is locally produced by EAT and cardiac cells, where it may exert autocrine and pro-fibrotic effects within the myocardium [197,198]. TMZ, a metabolic modulator used in ischemic heart disease and HF, has been shown to normalize circulating visfatin concentrations [199]. Considering the close association between EAT dysfunction and visfatin expression, TMZ may exert beneficial cardiometabolic effects partly through modulation of EAT-derived inflammatory signaling. These observations suggest a potential mechanistic link between trimetazidine therapy and EAT activity, warranting further investigation.
4. **MRA, ACEi, ARB, ARNI**. Renin–angiotensin–aldosterone system inhibitors, through their hormonal blockade, may play an important role in reducing EAT accumulation and its proinflammatory effects. Aldosterone is known to promote visceral and epicardial fat accumulation as well as adipose tissue inflammation, while activated renin-angiotensin system (RAS) and resultant production of angiotensin II is critically involved in obesity associated inflammation [200,201]. In an experimental mouse study using advanced magnetic resonance imaging techniques, eplerenone treatment reduced EAT volume and shifted its fatty acid composition toward greater unsaturation, which was associated with improved coronary microvascular function. Compared with untreated mice, eplerenone-treated animals showed lower saturated fatty acid fractions, reduced EAT mass, and improved myocardial perfusion reserve, suggesting beneficial effects on both adipose tissue metabolism and CV function [202]. Previous studies have shown that eplerenone can prevent and reverse obesity-related adipose inflammation and reduce CV death and HF hospitalization, especially in patients with abdominal obesity [203,204,205]. In the experimental study by Mitchell et al. was demonstrated that RAS inhibition reduces gene expression of inflammatory mediators in adipose tissue [206]. Furthermore, spironolactone and irbesartan appear to be more effective in patients with HFpEF and lower natriuretic peptide levels, possibly because these patients are more likely to have visceral adiposity and increased EAT [207,208]. These findings support the hypothesis that inhibition of aldosterone and RAS signaling may improve outcomes partly through modulation of visceral and epicardial adipose tissue activity.

Natriuretic peptide signaling also appears to have an important protective role in HFpEF by reducing adipose tissue inflammation, promoting adiponectin secretion, and limiting cardiac fibrosis [209,210,211]. However, obese patients with HFpEF often exhibit disproportionately low circulating natriuretic peptide levels, possibly due to pericardial restraint seen in excessive epicardial fat accumulation, enhanced neprilysin-mediated peptide degradation, and increased adipocyte clearance associated with hyperinsulinemia [64,212,213,214,215]. Since natriuretic peptides exert antiadipogenic effects, therapies that enhance natriuretic peptide activity, such as neprilysin inhibitors, may improve cardiac filling pressures and counteract the adverse effects of epicardial and visceral adiposity in HFpEF.

5. **Colchicine**. Colchicine exerts anti-inflammatory effects primarily through irreversible binding to α/β-tubulin dimers, thereby inhibiting microtubule polymerization. In neutrophils, where colchicine preferentially accumulates due to reduced P-glycoprotein–mediated efflux, this results in impaired endothelial adhesion, migration, and transmigration out of vessels. Colchicine also suppresses several inflammatory mediators, including TNF-α, leukotriene B4, prostaglandin E2, thromboxane A2, and cyclooxygenase-2 activity. In addition, it inhibits neutrophil α-defensin release and neutrophil–platelet aggregation, potentially reducing thrombotic burden. Another key mechanism is inhibition of the NLRP3 inflammasome, leading to reduced production of IL-1β and IL-18 and attenuation of innate immune activation [216]. Clinical data suggest that colchicine may also modulate EAT-related inflammation. In patients undergoing AF ablation, lower left atrial EAT volume was associated with reduced early AF recurrence during short-term colchicine treatment, indicating a potential interaction between EAT burden and the anti-inflammatory efficacy of colchicine after ablation [217]. Similarly, in the EKSTROM trial, patients with stable CAD treated with colchicine demonstrated a significant reduction in EAT volume on serial cardiac CT over 12 months compared with placebo, although no significant change in EAT density was observed. These findings suggest that colchicine may favorably influence EAT volume and provide possible mechanistic insight into its CV protective effects [218].

Given the central role of inflammatory pathways in EAT biology, other targeted anti-inflammatory therapies, including IL-1β, IL-6 and TNF-α inhibitors, may represent potential therapeutic strategies. However, their specific effect on EAT volume, activity and CV outcomes remains insufficiently studied and requires further mechanistic and clinical investigation.

## 7. Conclusions

EAT is heterogeneous by its nature, with initial metabolically favorable BAT activity contrasting the adverse cardiometabolic profile of white adipose tissue. Although excessive EAT is a common finding in T2DM individuals, little is known regarding the impact of EAT on early T2DM-related changes. These changes are attributable to the early pericardial disorder, which consists of both metabolic and hemodynamic changes. EAT could thus serve as a useful marker for risk assessment in T2DM individuals, as well as a potential target for contemporary and novel medical therapy. Moreover, EAT acts as a highly active perivascular adipose tissue depot with pronounced immunometabolic activity, contributing to a paradigm shift in the understanding of CAD pathophysiology from a predominantly endoluminal to an exoluminal perspective. Given that excessive EAT accumulation, oxidative stress, and low-grade inflammation are hallmarks of T2DM, it is crucial to act timely: „time is myocardium”, but timing may be EAT.

## Data Availability

The original contributions presented in this study are included in the article. Further inquiries can be directed to the corresponding authors.

## References

[1] Iacobellis G. (2022). Epicardial adipose tissue in contemporary cardiology. Nat. Rev. Cardiol..

[2] Kershaw E.E., Flier J.S. (2004). Adipose tissue as an endocrine organ. J. Clin. Endocrinol. Metab..

[3] Mazurek T., Zhang L., Zalewski A., Mannion J.D., Diehl J.T., Arafat H., Sarov-Blat L., O’Brien S., Keiper E.A., Johnson A.G. (2003). Human epicardial adipose tissue is a source of inflammatory mediators. Circulation.

[4] McAninch E.A., Fonseca T.L., Poggioli R., Panos A.L., Salerno T.A., Deng Y., Li Y., Bianco A.C., Iacobellis G. (2015). Epicardial adipose tissue has a unique transcriptome modified in severe coronary artery disease. Obesity.

[5] Iacobellis G., Willens H.J. (2009). Echocardiographic epicardial fat: A review of research and clinical applications. J. Am. Soc. Echocardiogr..

[6] Gaborit B., Venteclef N., Ancel P., Pelloux V., Gariboldi V., Leprince P., Amour J., Hatem S.N., Jouve E., Dutour A. (2015). Human epicardial adipose tissue has a specific transcriptomic signature depending on its anatomical peri-atrial, peri-ventricular, or peri-coronary location. Cardiovasc. Res..

[7] Zhao L., Guo Z., Wang P., Zheng M., Yang X., Liu Y., Ma Z., Chen M., Yang X. (2020). Proteomics of epicardial adipose tissue in patients with heart failure. J. Cell. Mol. Med..

[8] Camarena V., Sant D., Mohseni M., Salerno T., Zaleski M.L., Wang G., Iacobellis G. (2017). Novel atherogenic pathways from the differential transcriptome analysis of diabetic epicardial adipose tissue. Nutr. Metab. Cardiovasc. Dis..

[9] Zhang L., Zalewski A., Liu Y., Mazurek T., Cowan S., Martin J.L., Hofmann S.M., Vlassara H., Shi Y. (2003). Diabetes-induced oxidative stress and low-grade inflammation in porcine coronary arteries. Circulation.

[10] Wibmer A.G., Becher T., Eljalby M., Crane A., Andrieu P.C., Jiang C.S., Vaughan R., Schöder H., Cohen P. (2021). Brown adipose tissue is associated with healthier body fat distribution and metabolic benefits independent of regional adiposity. Cell Rep. Med..

[11] Vural B., Atalar F., Ciftci C., Demirkan A., Susleyici-Duman B., Gunay D., Akpinar B., Sagbas E., Ozbek U., Buyukdevrim A.S. (2008). Presence of fatty-acid-binding protein 4 expression in human epicardial adipose tissue in metabolic syndrome. Cardiovasc. Pathol..

[12] Ridker P.M., Rifai N., Stampfer M.J., Hennekens C.H. (2000). Plasma concentration of interleukin-6 and the risk of future myocardial infarction among apparently healthy men. Circulation.

[13] Ridker P.M., Rifai N., Pfeffer M., Sacks F., Lepage S., Braunwald E. (2000). Elevation of tumor necrosis factor-alpha and increased risk of recurrent coronary events after myocardial infarction. Circulation.

[14] Lindahl B., Toss H., Siegbahn A., Venge P., Wallentin L. (2000). Markers of myocardial damage and inflammation in relation to long-term mortality in unstable coronary artery disease. FRISC Study Group. Fragmin during Instability in Coronary Artery Disease. N. Engl. J. Med..

[15] Hotamisligil G.S., Arner P., Caro J.F., Atkinson R.L., Spiegelman B.M. (1995). Increased adipose tissue expression of tumor necrosis factor-alpha in human obesity and insulin resistance. J. Clin. Investig..

[16] Kern P.A., Ranganathan S., Li C., Wood L., Ranganathan G. (2001). Adipose tissue tumor necrosis factor and interleukin-6 expression in human obesity and insulin resistance. Am. J. Physiol. Endocrinol. Metab..

[17] Hauck A.K., Bernlohr D.A. (2016). Oxidative stress and lipotoxicity. J. Lipid Res..

[18] Ertunc M.E., Hotamisligil G.S. (2016). Lipid signaling and lipotoxicity in metaflammation: Indications for metabolic disease pathogenesis and treatment. J. Lipid Res..

[19] Chaurasia B., Summers S.A. (2015). Ceramides—Lipotoxic Inducers of Metabolic Disorders. Trends Endocrinol. Metab..

[20] Han J., Kaufman R.J. (2016). The role of ER stress in lipid metabolism and lipotoxicity. J. Lipid Res..

[21] Montgomery M.K., De Nardo W., Watt M.J. (2019). Impact of Lipotoxicity on Tissue “Cross Talk” and Metabolic Regulation. Physiology.

[22] Packer M. (2018). Epicardial Adipose Tissue May Mediate Deleterious Effects of Obesity and Inflammation on the Myocardium. J. Am. Coll. Cardiol..

[23] Cinti S., Mitchell G., Barbatelli G., Murano I., Ceresi E., Faloia E., Wang S., Fortier M., Greenberg A.S., Obin M.S. (2005). Adipocyte death defines macrophage localization and function in adipose tissue of obese mice and humans. J. Lipid Res..

[24] Strissel K.J., Stancheva Z., Miyoshi H., Perfield J.W., DeFuria J., Jick Z., Greenberg A.S., Obin M.S. (2007). Adipocyte death, adipose tissue remodeling, and obesity complications. Diabetes.

[25] Sommer G., Kralisch S., Stangl V., Vietzke A., Köhler U., Stepan H., Faber R., Schubert A., Lössner U., Bluher M. (2009). Secretory products from human adipocytes stimulate proinflammatory cytokine secretion from human endothelial cells. J. Cell. Biochem..

[26] Johnston E.K., Abbott R.D. (2023). Adipose Tissue Paracrine-, Autocrine-, and Matrix-Dependent Signaling during the Development and Progression of Obesity. Cells.

[27] Ludvik B., Nolan J.J., Baloga J., Sacks D., Olefsky J. (1995). Effect of obesity on insulin resistance in normal subjects and patients with NIDDM. Diabetes.

[28] Carey V.J., Walters E.E., Colditz G.A., Solomon C.G., Willett W.C., Rosner B.A., Speizer F.E., Manson J.E. (1997). Body fat distribution and risk of non-insulin-dependent diabetes mellitus in women. The Nurses’ Health Study. Am. J. Epidemiol..

[29] Chen L., Chen R., Wang H., Liang F. (2015). Mechanisms Linking Inflammation to Insulin Resistance. Int. J. Endocrinol..

[30] Hotamisligil G.S., Shargill N.S., Spiegelman B.M. (1993). Adipose expression of tumor necrosis factor-alpha: Direct role in obesity-linked insulin resistance. Science.

[31] Dandona P., Weinstock R., Thusu K., Abdel-Rahman E., Aljada A., Wadden T. (1998). Tumor necrosis factor-alpha in sera of obese patients: Fall with weight loss. J. Clin. Endocrinol. Metab..

[32] Mohallem R., Aryal U.K. (2020). Regulators of TNFα mediated insulin resistance elucidated by quantitative proteomics. Sci. Rep..

[33] Hirokawa J., Sakaue S., Furuya Y., Ishii J., Hasegawa A., Tagami S., Kawakami Y., Sakai M., Nishi S., Nishihira J. (1998). Tumor necrosis factor-alpha regulates the gene expression of macrophage migration inhibitory factor through tyrosine kinase-dependent pathway in 3T3-L1 adipocytes. J. Biochem..

[34] Libby P., Ridker P.M., Maseri A. (2002). Inflammation and atherosclerosis. Circulation.

[35] Azul L., Leandro A., Boroumand P., Klip A., Seiça R., Sena C.M. (2020). Increased inflammation, oxidative stress and a reduction in antioxidant defense enzymes in perivascular adipose tissue contribute to vascular dysfunction in type 2 diabetes. Free Radic. Biol. Med..

[36] Cosson E., Nguyen M.T., Rezgani I., Berkane N., Pinto S., Bihan H., Tatulashvili S., Taher M., Sal M., Soussan M. (2021). Epicardial adipose tissue volume and myocardial ischemia in asymptomatic people living with diabetes: A cross-sectional study. Cardiovasc. Diabetol..

[37] Parisi V., Rengo G., Perrone-Filardi P., Pagano G., Femminella G.D., Paolillo S., Petraglia L., Gambino G., Caruso A., Grimaldi M.G. (2016). Increased Epicardial Adipose Tissue Volume Correlates with Cardiac Sympathetic Denervation in Patients with Heart Failure. Circ. Res..

[38] White I.A. (2016). Cardiac Sympathetic Denervation in the Failing Heart: A Role for Epicardial Adipose Tissue. Circ. Res..

[39] Mazurek T., Opolski G. (2015). Pericoronary adipose tissue: A novel therapeutic target in obesity-related coronary atherosclerosis. J. Am. Coll. Nutr..

[40] Hao S., Sui X., Wang J., Zhang J., Pei Y., Guo L., Liang Z. (2021). Secretory products from epicardial adipose tissue induce adverse myocardial remodeling after myocardial infarction by promoting reactive oxygen species accumulation. Cell Death Dis..

[41] Carena M.C., Badi I., Polkinghorne M., Akoumianakis I., Psarros C., Wahome E., Kotanidis C.P., Akawi N., Antonopoulos A.S., Chauhan J. (2023). Role of Human Epicardial Adipose Tissue-Derived miR-92a-3p in Myocardial Redox State. J. Am. Coll. Cardiol..

[42] Haemers P., Hamdi H., Guedj K., Suffee N., Farahmand P., Popovic N., Claus P., LePrince P., Nicoletti A., Jalife J. (2017). Atrial fibrillation is associated with the fibrotic remodelling of adipose tissue in the subepicardium of human and sheep atria. Eur. Heart J..

[43] Nalliah C.J., Bell J.R., Raaijmakers A.J.A., Waddell H.M., Wells S.P., Bernasochi G.B., Montgomery M.K., Binny S., Watts T., Joshi S.B. (2020). Epicardial Adipose Tissue Accumulation Confers Atrial Conduction Abnormality. J. Am. Coll. Cardiol..

[44] Meulendijks E.R., Al-Shama R.F.M., Kawasaki M., Fabrizi B., Neefs J., Wesselink R., Ernault A.C., Piersma S., Pham T.V., Jimenez C.R. (2023). Atrial epicardial adipose tissue abundantly secretes myeloperoxidase and activates atrial fibroblasts in patients with atrial fibrillation. J. Transl. Med..

[45] Oba K., Maeda M., Maimaituxun G., Yamaguchi S., Arasaki O., Fukuda D., Yagi S., Hirata Y., Nishio S., Iwase T. (2018). Effect of the Epicardial Adipose Tissue Volume on the Prevalence of Paroxysmal and Persistent Atrial Fibrillation. Circ. J..

[46] Schram-Serban C., Heida A., Roos-Serote M.C., Knops P., Kik C., Brundel B., Bogers A.J.J.C., de Groot N.M.S. (2020). Heterogeneity in Conduction Underlies Obesity-Related Atrial Fibrillation Vulnerability. Circ. Arrhythmia Electrophysiol..

[47] Bekar L., Kalçık M., Çelik O., Alp Ç., Yetim M., Doğan T., Ekinözü İ., Karaarslan O., Çamkıran V., Karavelioğlu Y. (2019). Presence of fragmented QRS is associated with increased epicardial adipose tissue thickness in hypertensive patients. J. Clin. Ultrasound.

[48] Shen J., Zhu D., Chen L., Cang J., Zhao Z., Ji Y., Liu S., Miao H., Liu Y., Zhou Q. (2023). Relationship between epicardial adipose tissue measured by computed tomography and premature ventricular complexes originating from different sites. Europace.

[49] Ernault A.C., Meijborg V.M.F., Coronel R. (2021). Modulation of Cardiac Arrhythmogenesis by Epicardial Adipose Tissue: JACC State-of-the-Art Review. J. Am. Coll. Cardiol..

[50] Kankaanpää M., Lehto H.R., Pärkkä J.P., Komu M., Viljanen A., Ferrannini E., Knuuti J., Nuutila P., Parkkola R., Iozzo P. (2006). Myocardial triglyceride content and epicardial fat mass in human obesity: Relationship to left ventricular function and serum free fatty acid levels. J. Clin. Endocrinol. Metab..

[51] Sepehri Shamloo A., Schoene K., Stauber A., Darma A., Dagres N., Dinov B., Bertagnolli L., Hilbert S., Müssigbrodt A., Husser D. (2019). Epicardial adipose tissue thickness as an independent predictor of ventricular tachycardia recurrence following ablation. Heart Rhythm.

[52] Grubić Rotkvić P., Planinić Z., Liberati Pršo A.M., Šikić J., Galić E., Rotkvić L. (2021). The Mystery of Diabetic Cardiomyopathy: From Early Concepts and Underlying Mechanisms to Novel Therapeutic Possibilities. Int. J. Mol. Sci..

[53] Seferović P.M., Paulus W.J., Rosano G., Polovina M., Petrie M.C., Jhund P.S., Tschöpe C., Sattar N., Piepoli M., Papp Z. (2024). Diabetic myocardial disorder. A clinical consensus statement of the Heart Failure Association of the ESC and the ESC Working Group on Myocardial & Pericardial Diseases. Eur. J. Heart Fail..

[54] Pop-Busui R., Januzzi J.L., Bruemmer D., Butalia S., Green J.B., Horton W.B., Knight C., Levi M., Rasouli N., Richardson C.R. (2022). Heart Failure: An Underappreciated Complication of Diabetes. A Consensus Report of the American Diabetes Association. Diabetes Care.

[55] Kenny H.C., Abel E.D. (2019). Heart Failure in Type 2 Diabetes Mellitus. Circ. Res..

[56] Bugger H., Abel E.D. (2014). Molecular mechanisms of diabetic cardiomyopathy. Diabetologia.

[57] Jørgensen P.G., Jensen M.T., Biering-Sørensen T., Mogelvang R., Fritz-Hansen T., Vilsbøll T., Rossing P., Jensen J.S. (2018). Burden of Uncontrolled Metabolic Risk Factors and Left Ventricular Structure and Function in Patients with Type 2 Diabetes Mellitus. J. Am. Heart Assoc..

[58] Li Y., Liu B., Li Y., Jing X., Deng S., Yan Y., She Q. (2019). Epicardial fat tissue in patients with diabetes mellitus: A systematic review and meta-analysis. Cardiovasc. Diabetol..

[59] Đuzel Čokljat A., Grubić Rotkvić P., Čokljat D., Ferri Certić J., Babić Z. (2025). Epicardial adipose tissue in diabetic myocardial disorder: Role of echocardiography. World J. Diabetes.

[60] van Woerden G., van Veldhuisen D.J., Westenbrink B.D., de Boer R.A., Rienstra M., Gorter T.M. (2022). Connecting epicardial adipose tissue and heart failure with preserved ejection fraction: Mechanisms, management and modern perspectives. Eur. J. Heart Fail..

[61] Gorter T.M., van Woerden G., Rienstra M., Dickinson M.G., Hummel Y.M., Voors A.A., Hoendermis E.S., van Veldhuisen D.J. (2020). Epicardial Adipose Tissue and Invasive Hemodynamics in Heart Failure with Preserved Ejection Fraction. JACC Heart Fail..

[62] Koepp K.E., Obokata M., Reddy Y.N.V., Olson T.P., Borlaug B.A. (2020). Hemodynamic and Functional Impact of Epicardial Adipose Tissue in Heart Failure with Preserved Ejection Fraction. JACC Heart Fail..

[63] Oduah M.T., Sundaram V., Reddy Y.N. (2023). Epicardial Fat in Heart Failure with Preserved Ejection Fraction: Bad Actor or Just Lying Around?. Card. Fail. Rev..

[64] Obokata M., Reddy Y.N.V., Pislaru S.V., Melenovsky V., Borlaug B.A. (2017). Evidence Supporting the Existence of a Distinct Obese Phenotype of Heart Failure with Preserved Ejection Fraction. Circulation.

[65] Ng A.C., Delgado V., Bertini M., van der Meer R.W., Rijzewijk L.J., Shanks M., Nucifora G., Smit J.W., Diamant M., Romijn J.A. (2009). Findings from left ventricular strain and strain rate imaging in asymptomatic patients with type 2 diabetes mellitus. Am. J. Cardiol..

[66] Choy M., Huang Y., Peng Y., Liang W., He X., Chen C., Li J., Zhu W., Wei F.F., Dong Y. (2023). Association between epicardial adipose tissue and incident heart failure mediating by alteration of natriuretic peptide and myocardial strain. BMC Med..

[67] Li N., Cao Y., Li Y., Zhang K., Zhang L., Luo Q., Sun W., Shi H. (2025). Predictive value of epicardial adipose tissue volume for early detection of left ventricular dysfunction in patients suspected of coronary artery disease. Clin. Radiol..

[68] Schulz A., Backhaus S.J., Lange T., Evertz R., Kutty S., Kowallick J.T., Hasenfuß G., Schuster A. (2024). Impact of epicardial adipose tissue on cardiac function and morphology in patients with diastolic dysfunction. ESC Heart Fail..

[69] Yang M.C., Liu H.K., Tsai C.C., Su Y.T., Wu J.R. (2022). Epicardial Adipose Tissue Was Highly Associated with Reduction in Left Ventricular Diastolic Function as Early as in Adolescence. Acta Cardiol. Sin..

[70] Topuz M., Dogan A. (2017). The effect of epicardial adipose tissue thickness on left ventricular diastolic functions in patients with normal coronary arteries. Kardiol. Pol..

[71] de Wit-Verheggen V.H.W., Altintas S., Spee R.J.M., Mihl C., van Kuijk S.M.J., Wildberger J.E., Schrauwen-Hinderling V.B., Kietselaer B.L.J.H., van de Weijer T. (2020). Pericardial fat and its influence on cardiac diastolic function. Cardiovasc. Diabetol..

[72] Setti M., Benfari G., Mele D., Rossi A., Ballo P., Galderisi M., Henein M., Nistri S. (2020). Discrepancies in Assessing Diastolic Function in Pre-Clinical Heart Failure Using Different Algorithms-A Primary Care Study. Diagnostics.

[73] Ishikawa H., Sugiyama T., Otsuka K., Yamaura H., Hojo K., Kono Y., Ito A., Yamazaki T., Shimada K., Kasayuki N. (2024). Impact of epicardial adipose tissue on diastolic dysfunction in patients with chronic coronary syndrome and preserved left ventricular ejection fraction. Eur. Heart J.-Imaging Methods Pract..

[74] Mancio J., Azevedo D., Fragao-Marques M., Falcao-Pires I., Leite-Moreira A., Lunet N., Fontes-Carvalho R., Bettencourt N. (2019). Meta-Analysis of Relation of Epicardial Adipose Tissue Volume to Left Atrial Dilation and to Left Ventricular Hypertrophy and Functions. Am. J. Cardiol..

[75] Christensen R.H., Hansen C.S., von Scholten B.J., Jensen M.T., Pedersen B.K., Schnohr P., Vilsbøll T., Rossing P., Jørgensen P.G. (2019). Epicardial and pericardial adipose tissues are associated with reduced diastolic and systolic function in type 2 diabetes. Diabetes Obes. Metab..

[76] Nakazato R., Rajani R., Cheng V.Y., Shmilovich H., Nakanishi R., Otaki Y., Gransar H., Slomka P.J., Hayes S.W., Thomson L.E. (2012). Weight change modulates epicardial fat burden: A 4-year serial study with non-contrast computed tomography. Atherosclerosis.

[77] Willens H.J., Byers P., Chirinos J.A., Labrador E., Hare J.M., de Marchena E. (2007). Effects of weight loss after bariatric surgery on epicardial fat measured using echocardiography. Am. J. Cardiol..

[78] Graziani F., Leone A.M., Cialdella P., Basile E., Pennestri F., Della Bona R., Iaconelli A., Liuzzo G., Biasucci L.M., Cardillo M.T. (2013). Effects of bariatric surgery on cardiac remodeling: Clinical and pathophysiologic implications. Int. J. Cardiol..

[79] Henry J.A., Abdesselam I., Deal O., Lewis A.J., Rayner J., Bernard M., Dutour A., Gaborit B., Kober F., Soghomonian A. (2023). Changes in epicardial and visceral adipose tissue depots following bariatric surgery and their effect on cardiac geometry. Front. Endocrinol..

[80] Asteria C., Secchi F., Morricone L., Malavazos A.E., Francesconi S., Milani V., Giovanelli A. (2025). Open-bore MRI Scanner Assessment of Epicardial Adipose Tissue after Bariatric Surgery: A Pilot Study. Endocr. Metab. Immune Disord. Drug Targets.

[81] Hunt S.C., Davidson L.E., Adams T.D., Ranson L., McKinlay R.D., Simper S.C., Litwin S.E. (2021). Associations of Visceral, Subcutaneous, Epicardial, and Liver Fat with Metabolic Disorders up to 14 Years After Weight Loss Surgery. Metab. Syndr. Relat. Disord..

[82] Mikamo H., Jiang M., Noro M., Suzuki Y., Hiruta N., Unoki-Kubota H., Schneider W.J., Bujo H. (2018). Susceptibilities of epicardial and subcutaneous fat tissue for browning-gene expression and diet-induced volume reduction are different. Mol. Med. Rep..

[83] Kuleta K., Krauz K., Żmuda J., Momot K., Zarębiński M., Poprawa I., Wojciechowska M. (2025). Pharmacological and Non-Pharmacological Interventions in Diabetes Mellitus: Effects on Epicardial Adipose Tissue. Int. J. Mol. Sci..

[84] Visseren F.L.J., Mach F., Smulders Y.M., Carballo D., Koskinas K.C., Bäck M., Benetos A., Biffi A., Boavida J.-M., Capodanno D. (2021). 2021 ESC guidelines on cardiovascular disease prevention in clinical practice. Eur. Heart J..

[85] Barrio-Lopez M.T., Ruiz-Canela M., Goni L., Valiente A.M., Garcia S.R., de la O V., Anton B.D., Fernandez-Friera L., Castellanos E., Martínez-González M.A. (2024). Mediterranean diet and epicardial adipose tissue in patients with atrial fibrillation treated with ablation: A substudy of the ‘PREDIMAR’ trial. Eur. J. Prev. Cardiol..

[86] Derosa G., D’Angelo A., Angelini F., Belli L., Cicero A.F.G., Da Ros R., De Pergola G., Gaudio G.V., Lupi A., Sartore G. (2024). Nutraceuticals and Supplements in Management of Prediabetes and Diabetes. Nutrients.

[87] Huang Q., Zhan J., Gui Y., Ma M., Li E. (2026). Effects of different vitamin D supplements on body fat distribution and glucolipid metabolism in patients with obesity-associated metabolic syndrome: A meta-analysis. Medicine.

[88] Christensen R.H., Wedell-Neergaard A.S., Lehrskov L.L., Legaard G.E., Dorph E., Larsen M.K., Launbo N., Fagerlind S.R., Seide S.K., Nymand S. (2019). Effect of Aerobic and Resistance Exercise on Cardiac Adipose Tissues: Secondary Analyses from a Randomized Clinical Trial. JAMA Cardiol..

[89] Wilund K.R., Tomayko E.J., Wu P.T., Ryong Chung H., Vallurupalli S., Lakshminarayanan B., Fernhall B. (2010). Intradialytic exercise training reduces oxidative stress and epicardial fat: A pilot study. Nephrol. Dial. Transplant..

[90] Rosety M.A., Pery M.T., Rodriguez-Pareja M.A., Diaz A., Rosety J., Garcia N., Brenes-Martin F., Rosety-Rodriguez M., Toro R., Ordonez F.J. (2015). A Short-Term Circuit Resistance Programme Reduced Epicardial Fat in Obese Aged Women. Nutr. Hosp..

[91] Kim M.K., Tomita T., Kim M.J., Sasai H., Maeda S., Tanaka K. (2009). Aerobic exercise training reduces epicardial fat in obese men. J. Appl. Physiol..

[92] Zhou J., Gao X., Zhang D., Jiang C., Yu W. (2025). Effects of breaking up prolonged sitting via exercise snacks intervention on the body composition and plasma metabolomics of sedentary obese adults: A randomized controlled trial. Endocr. J..

[93] Honkala S.M., Motiani K.K., Eskelinen J.J., Savolainen A., Saunavaara V., Virtanen K.A., Loyttyniemi E., Kapanen J., Knuuti J., Kalliokoski K.K. (2017). Exercise Training Reduces Intrathoracic Fat Regardless of Defective Glucose Tolerance. Med. Sci. Sports Exerc..

[94] Milanese G., Silva M., Bruno L., Goldoni M., Benedetti G., Rossi E., Ferrari C., Grutta L., Maffei E., Toia P. (2019). Quantification of epicardial fat with cardiac CT angiography and association with cardiovascular risk factors in symptomatic patients: From the ALTER-BIO registry. Diagn. Interv. Radiol..

[95] Monti M., Monti A., Murdolo G., Di Renzi P., Pirro M.R., Borgognoni F., Vincentelli G.M. (2014). Correlation between epicardial fat and cigarette smoking: CT imaging in patients with metabolic syndrome. Scand. Cardiovasc. J..

[96] Gac P., Czerwinska K., Poreba M., Macek P., Mazur G., Poreba R. (2021). Environmental Tobacco Smoke Exposure Estimated Using the SHSES Scale and Epicardial Adipose Tissue Thickness in Hypertensive Patients. Cardiovasc. Toxicol..

[97] Messineo L., Bakker J.P., Cronin J., Yee J., White D.P. (2024). Obstructive sleep apnea and obesity: A review of epidemiology, pathophysiology and the effect of weight-loss treatments. Sleep Med. Rev..

[98] Young T., Peppard P.E., Gottlieb D.J. (2002). Epidemiology of obstructive sleep apnea. Am. J. Respir. Crit. Care Med..

[99] Varghese R.T., Akurati S., Iacobellis G. (2025). Epicardial fat and sleep apnea: Perspective mechanisms, diagnostics, and therapeutics. Obes. Endocrinol..

[100] Parekh A. (2024). Hypoxic burden: Definitions, pathophysiological concepts, methods of evaluation, and clinical relevance. Curr. Opin. Pulm. Med..

[101] Beaudin A.E., Hanly P.J., Raneri J.K., Younes M., Pun M., Anderson T.J., Poulin M.J. (2022). Impact of intermittent hypoxia on human vascular responses during sleep. Exp. Neurol..

[102] Adeva-Andany M.M., Domínguez-Montero A., Castro-Quintela E., Funcasta-Calderón R., Fernández-Fernández C. (2024). Hypoxia-Induced Insulin Resistance Mediates the Elevated Cardiovascular Risk in Patients with Obstructive Sleep Apnea: A Comprehensive Review. Rev. Cardiovasc. Med..

[103] Kostopoulos K., Alhanatis E., Pampoukas K., Georgiopoulos G., Zourla A., Panoutsopoulos A., Kallianos A., Velentza L., Zarogoulidis P., Trakada G. (2016). CPAP therapy induces favorable short-term changes in epicardial fat thickness and vascular and metabolic markers in OSAHS. Sleep. Breath..

[104] Palanisamy S., Yien E.L.H., Shi L.W., Si L.Y., Qi S.H., Ling L.S.C., Lun T.W., Chen Y.N. (2018). Systematic Review of Efficacy and Safety of Newer Antidiabetic Drugs Approved from 2013 to 2017 in Controlling HbA1c in Diabetes Patients. Pharmacy.

[105] Wang C.P., Hsu H.L., Hung W.C., Yu T.H., Chen Y.H., Chiu C.A., Lu L.F., Chung F.M., Shin S.J., Lee Y.J. (2009). Increased epicardial adipose tissue (EAT) volume in type 2 diabetes mellitus and association with metabolic syndrome and severity of coronary atherosclerosis. Clin. Endocrinol..

[106] Philouze C., Obert P., Nottin S., Benamor A., Barthez O., Aboukhoudir F. (2018). Dobutamine Stress Echocardiography Unmasks Early Left Ventricular Dysfunction in Asymptomatic Patients with Uncomplicated Type 2 Diabetes: A Comprehensive Two-Dimensional Speckle-Tracking Imaging Study. J. Am. Soc. Echocardiogr..

[107] Altara R., Giordano M., Nordén E.S., Cataliotti A., Kurdi M., Bajestani S.N., Booz G.W. (2017). Targeting Obesity and Diabetes to Treat Heart Failure with Preserved Ejection Fraction. Front. Endocrinol..

[108] Udell J.A., Cavender M.A., Bhatt D.L., Chatterjee S., Farkouh M.E., Scirica B.M. (2015). Glucose lowering drugs or strategies and cardiovascular outcomes in patients with or at risk for type 2 diabetes: A meta-analysis of randomized controlled trials. Lancet Diabetes Endocrinol..

[109] Cignarelli A., Giorgino F., Vettor R. (2013). Pharmacologic agents for type 2 diabetes therapy and regulation of adipogenesis. Arch. Physiol. Biochem..

[110] Bailey C.J., Turner R.C. (1996). Metformin. N. Engl. J. Med..

[111] Emslie-Smith A.M., Boyle D.I., Evans J.M., Sullivan F., Morris A.D., DARTS/MEMO Collaboration (2001). Contraindications to metformin therapy in patients with Type 2 diabetes—A population-based study of adherence to prescribing guidelines. Diabet. Med..

[112] Ziyrek M., Kahraman S., Ozdemir E., Dogan A. (2019). Metformin monotherapy significantly decreases epicardial adipose tissue thickness in newly diagnosed type 2 diabetes patients. Rev. Port. Cardiol..

[113] Gunes H., Gunes H., Ozmen S., Celik E., Temiz F. (2020). Effects of metformin on epicardial adipose tissue and atrial electromechanical delay of obese children with insulin resistance. Cardiol. Young.

[114] Li B., Po S.S., Zhang B., Bai F., Li J., Qin F., Liu N., Sun C., Xiao Y., Tu T. (2020). Metformin regulates adiponectin signalling in epicardial adipose tissue and reduces atrial fibrillation vulnerability. J. Cell. Mol. Med..

[115] Elia E.M., Pustovrh C., Amalfi S., Devoto L., Motta A.B. (2011). Link between metformin and the peroxisome proliferator-activated receptor gamma pathway in the uterine tissue of hyperandrogenized prepubertal mice. Fertil. Steril..

[116] Sun L., Xu Y.W., Han J., Liang H., Wang N., Cheng Y. (2015). 12/15-Lipoxygenase metabolites of arachidonic acid activate PPARγ: A possible neuroprotective effect in ischemic brain. J. Lipid Res..

[117] Mansour H.H., El Kiki S.M., Galal S.M. (2017). Metformin and low dose radiation modulates cisplatin-induced oxidative injury in rat via PPAR-gamma and MAPK pathways. Arch. Biochem. Biophys..

[118] Su J.R., Lu Z.H., Su Y., Zhao N., Dong C.L., Sun L., Zhao S.F., Li Y. (2016). Relationship of Serum Adiponectin Levels and Metformin Therapy in Patients with Type 2 Diabetes. Horm. Metab. Res..

[119] Evia-Viscarra M.L., Rodea-Montero E.R., Apolinar-Jimenez E., Munoz-Noriega N., Garcia-Morales L.M., Leanos-Perez C., Figueroa-Barron M., Sanchez-Fierros D., Reyes-Garcia J.G. (2012). The effects of metformin on inflammatory mediators in obese adolescents with insulin resistance: Controlled randomized clinical trial. J. Pediatr. Endocrinol. Metab..

[120] Iacobellis G., Mohseni M., Bianco S.D., Banga P.K. (2017). Liraglutide causes large and rapid epicardial fat reduction. Obesity.

[121] Dormandy J.A., Charbonnel B., Eckland D.J., Erdmann E., Massi-Benedetti M., Moules I.K., Skene A.M., Tan M.H., Lefèbvre P.J., Murray G.D. (2005). PROactive Investigators. Secondary prevention of macrovascular events in patients with type 2 diabetes in the PROactive Study (PROspective pioglitAzone Clinical Trial In macroVascular Events): A randomised controlled trial. Lancet.

[122] Filipova E., Uzunova K., Kalinov K., Vekov T. (2017). Effects of pioglitazone therapy on blood parameters, weight and BMI: A meta-analysis. Diabetol. Metab. Syndr..

[123] Zhang F., Pan X., Zhang X., Tong N. (2024). The effect of thiazolidinediones on body fat redistribution in adults: A systematic review and meta-analysis of randomized controlled trials. Obes. Rev..

[124] Moody A.J., Molina-Wilkins M., Clarke G.D., Merovci A., Solis-Herrera C., Cersosimo E., Chilton R.J., Iozzo P., Gastaldelli A., Abdul-Ghani M. (2023). Pioglitazone reduces epicardial fat and improves diastolic function in patients with type 2 diabetes. Diabetes Obes. Metab..

[125] Jonker J.T., Lamb H.J., van der Meer R.W., Rijzewijk L.J., Menting L.J., Diamant M., Bax J.J., de Roos A., Romijn J.A., Smit J.W. (2010). Pioglitazone compared with metformin increases pericardial fat volume in patients with type 2 diabetes mellitus. J. Clin. Endocrinol. Metab..

[126] Sacks H.S., Fain J.N., Cheema P., Bahouth S.W., Garrett E., Wolf R.Y., Wolford D., Samaha J. (2011). Inflammatory genes in epicardial fat contiguous with coronary atherosclerosis in the metabolic syndrome and type 2 diabetes: Changes associated with pioglitazone. Diabetes Care.

[127] Distel E., Penot G., Cadoudal T., Balguy I., Durant S., Benelli C. (2012). Early induction of a brown-like phenotype by rosiglitazone in the epicardial adipose tissue of fatty Zucker rats. Biochimie.

[128] Zhu J., Yu X., Zheng Y., Li J., Wang Y., Lin Y., He Z., Zhao W., Chen C., Qiu K. (2020). Association of glucose-lowering medications with cardiovascular outcomes: An umbrella review and evidence map. Lancet Diabetes Endocrinol..

[129] Karagiannis T., Paschos P., Paletas K., Matthews D.R., Tsapas A. (2012). Dipeptidyl peptidase-4 inhibitors for treatment of type 2 diabetes mellitus in the clinical setting: Systematic review and meta-analysis. BMJ.

[130] ElSayed N.A., Aleppo G., Aroda V.R., Bannuru R.R., Brown F.M., Bruemmer D., Collins B.S., Das S.R., Hilliard M.E., Isaacs D. (2023). 10. Cardiovascular Disease and Risk Management: Standards of Care in Diabetes-2023. Diabetes Care.

[131] Lima-Martinez M.M., Paoli M., Rodney M., Balladares N., Contreras M., D’Marco L., Iacobellis G. (2016). Effect of sitagliptin on epicardial fat thickness in subjects with type 2 diabetes and obesity: A pilot study. Endocrine.

[132] Hiruma S., Shigiyama F., Hisatake S., Mizumura S., Shiraga N., Hori M., Ikeda T., Hirose T., Kumashiro N. (2021). A prospective randomized study comparing effects of empagliflozin to sitagliptin on cardiac fat accumulation, cardiac function, and cardiac metabolism in patients with early-stage type 2 diabetes: The ASSET study. Cardiovasc. Diabetol..

[133] Hirose M., Takano H., Hasegawa H., Tadokoro H., Hashimoto N., Takemura G., Kobayashi Y. (2017). The effects of dipeptidyl peptidase-4 on cardiac fibrosis in pressure overload-induced heart failure. J. Pharmacol. Sci..

[134] Mulvihill E.E., Varin E.M., Ussher J.R., Campbell J.E., Bang K.W., Abdullah T., Baggio L.L., Drucker D.J. (2016). Inhibition of Dipeptidyl Peptidase-4 Impairs Ventricular Function and Promotes Cardiac Fibrosis in High Fat-Fed Diabetic Mice. Diabetes.

[135] Palmer S.C., Tendal B., Mustafa R.A., Vandvik P.O., Li S., Hao Q., Tunnicliffe D., Ruospo M., Natale P., Saglimbene V. (2021). Sodium-glucose cotransporter protein-2 (SGLT-2) inhibitors and glucagon-like peptide-1 (GLP-1) receptor agonists for type 2 diabetes: Systematic review and network meta-analysis of randomised controlled trials. BMJ.

[136] Malavazos A.E., Iacobellis G., Dozio E., Basilico S., Di Vincenzo A., Dubini C., Menicanti L., Vianello E., Meregalli C., Ruocco C. (2023). Human epicardial adipose tissue expresses glucose-dependent insulinotropic polypeptide, glucagon, and glucagon-like peptide-1 receptors as potential targets of pleiotropic therapies. Eur. J. Prev. Cardiol..

[137] Iacobellis G., Camarena V., Sant D.W., Wang G. (2017). Human Epicardial Fat Expresses Glucagon-Like Peptide 1 and 2 Receptors Genes. Horm. Metab. Res..

[138] Morano S., Romagnoli E., Filardi T., Nieddu L., Mandosi E., Fallarino M., Turinese I., Dagostino M.P., Lenzi A., Carnevale V. (2015). Short-term effects of glucagon-like peptide 1 (GLP-1) receptor agonists on fat distribution in patients with type 2 diabetes mellitus: An ultrasonography study. Acta Diabetol..

[139] Iacobellis G., Villasante Fricke A.C. (2020). Effects of Semaglutide Versus Dulaglutide on Epicardial Fat Thickness in Subjects with Type 2 Diabetes and Obesity. J. Endocr. Soc..

[140] Myasoedova V.A., Parisi V., Moschetta D., Valerio V., Conte M., Massaiu I., Bozzi M., Celeste F., Leosco D., Iaccarino G. (2023). Efficacy of cardiometabolic drugs in reduction of epicardial adipose tissue: A systematic review and meta-analysis. Cardiovasc. Diabetol..

[141] Dozio E., Vianello E., Malavazos A.E., Tacchini L., Schmitz G., Iacobellis G., Corsi Romanelli M.M. (2019). Epicardial adipose tissue GLP-1 receptor is associated with genes involved in fatty acid oxidation and white-to-brown fat differentiation: A target to modulate cardiovascular risk?. Int. J. Cardiol..

[142] Hong J.Y., Park K.Y., Kim B.J., Hwang W.M., Kim D.H., Lim D.M. (2016). Effects of Short-Term Exenatide Treatment on Regional Fat Distribution, Glycated Hemoglobin Levels, and Aortic Pulse Wave Velocity of Obese Type 2 Diabetes Mellitus Patients. Endocrinol. Metab..

[143] Vlachopoulos C., Aznaouridis K., Stefanadis C. (2010). Prediction of cardiovascular events and all-cause mortality with arterial stiffness: Asystematic review and meta-analysis. J. Am. Coll. Cardiol..

[144] Nguyen T.D., Shingu Y., Amorim P.A., Schenkl C., Schwarzer M., Doenst T. (2018). GLP-1 Improves Diastolic Function and Survival in Heart Failure with Preserved Ejection Fraction. J. Cardiovasc. Transl. Res..

[145] Hummel C.S., Lu C., Loo D.D., Hirayama B.A., Voss A.A., Wright E.M. (2011). Glucose transport by human renal Na+/D-glucose cotransporters SGLT1 and SGLT2. Am. J. Physiol. Cell Physiol..

[146] Grubić Rotkvić P., Cigrovski Berković M., Bulj N., Rotkvić L., Ćelap I. (2020). Sodium-glucose cotransporter 2 inhibitors’ mechanisms of action in heart failure. World J. Diabetes.

[147] Cinti F., Leccisotti L., Sorice G.P., Capece U., D’Amario D., Lorusso M., Gugliandolo S., Morciano C., Guarneri A., Guzzardi M.A. (2023). Dapagliflozin treatment is associated with a reduction of epicardial adipose tissue thickness and epicardial glucose uptake in human type 2 diabetes. Cardiovasc. Diabetol..

[148] Diaz-Rodriguez E., Agra R.M., Fernandez A.L., Adrio B., Garcia-Caballero T., Gonzalez-Juanatey J.R., Eiras S. (2018). Effects of dapagliflozin on human epicardial adipose tissue: Modulation of insulin resistance, inflammatory chemokine production, and differentiation ability. Cardiovasc. Res..

[149] Sato T., Aizawa Y., Yuasa S., Kishi S., Fuse K., Fujita S., Ikeda Y., Kitazawa H., Takahashi M., Sato M. (2018). The effect of dapagliflozin treatment on epicardial adipose tissue volume. Cardiovasc. Diabetol..

[150] Bao Y., Hu Y., Shi M., Zhao Z. (2025). SGLT2 inhibitors reduce epicardial adipose tissue more than GLP-1 agonists or exercise interventions in patients with type 2 diabetes mellitus and/or obesity: A systematic review and network meta-analysis. Diabetes Obes. Metab..

[151] Verma S., McMurray J.J.V. (2018). SGLT2 inhibitors and mechanisms of cardiovascular benefit: A state-of-the-art review. Diabetologia.

[152] Lau D.C., Dhillon B., Yan H., Szmitko P.E., Verma S. (2005). Adipokines: Molecular links between obesity and atheroslcerosis. Am. J. Physiol. Heart Circ. Physiol..

[153] Garvey W.T., Van Gaal L., Leiter L.A., Vijapurkar U., List J., Cuddihy R., Ren J., Davies M.J. (2018). Effects of canagliflozin versus glimepiride on adipokines and inflammatory biomarkers in type 2 diabetes. Metabolism.

[154] Hirst J.A., Farmer A.J., Dyar A., Lung T.W., Stevens R.J. (2013). Estimating the effect of sulfonylurea on HbA1c in diabetes: A systematic review and meta-analysis. Diabetologia.

[155] Forst T., Hanefeld M., Jacob S., Moeser G., Schwenk G., Pfützner A., Haupt A. (2013). Association of sulphonylurea treatment with all-cause and cardiovascular mortality: A systematic review and meta-analysis of observational studies. Diabetes Vasc. Dis. Res..

[156] Gerich J., Raskin P., Jean-Louis L., Purkayastha D., Baron M.A. (2005). PRESERVE-beta: Two-year efficacy and safety of initial combination therapy with nateglinide or glyburide plus metformin. Diabetes Care.

[157] Petersen M.C., Shulman G.I. (2018). Mechanisms of Insulin Action and Insulin Resistance. Physiol. Rev..

[158] Dimitriadis G., Mitrou P., Lambadiari V., Maratou E., Raptis S.A. (2011). Insulin effects in muscle and adipose tissue. Diabetes Res. Clin. Pract..

[159] Nichols G.A., Koro C.E., Gullion C.M., Ephross S.A., Brown J.B. (2005). The incidence of congestive heart failure associated with antidiabetic therapies. Diabetes Metab. Res. Rev..

[160] Marchington J., Pond C. (1990). Site-specific properties of pericardial and epicardial adipose tissue: The effects of insulin and high-fat feeding on lipogenesis and the incorporation of fatty acids in vitro. Int. J. Obes..

[161] Trabzon G., Güngör Ş., Güllü Ş.D., Çalışkan O.F., Güllü U.U. (2025). Evaluation of epicardial adipose tissue in children with type 1 diabetes. Pediatr. Res..

[162] Tiwari S., Riazi S., Ecelbarger C.A. (2007). Insulin’s impact on renal sodium transport and blood pressure in health, obesity, and diabetes. Am. J. Physiol. Ren. Physiol..

[163] Elisha B., Azar M., Taleb N., Bernard S., Iacobellis G., Rabasa-Lhoret R. (2016). Body Composition and Epicardial Fat in Type 2 Diabetes Patients Following Insulin Detemir Versus Insulin Glargine Initiation. Horm. Metab. Res..

[164] Soucek F., Covassin N., Singh P., Ruzek L., Kara T., Suleiman M., Lerman A., Koestler C., Friedman P.A., Lopez-Jimenez F. (2015). Effects of atorvastatin (80 mg) therapy on quantity of epicardial adipose tissue in patients undergoing pulmonary vein isolation for atrial fibrillation. Am. J. Cardiol..

[165] Cho K.I., Kim B.J., Cha T.J., Heo J.H., Kim H.S., Lee J.W. (2015). Impact of duration and dosage of statin treatment and epicardial fat thickness on the recurrence of atrial fibrillation after electrical cardioversion. Heart Vessel..

[166] Yamada Y., Takeuchi S., Yoneda M., Ito S., Sano Y., Nagasawa K., Matsuura N., Uchinaka A., Murohara T., Nagata K. (2017). Atorvastatin reduces cardiac and adipose tissue inflammation in rats with metabolic syndrome. Int. J. Cardiol..

[167] Alexopoulos N., Melek B.H., Arepalli C.D., Hartlage G.R., Chen Z., Kim S., Stillman A.E., Raggi P. (2013). Effect of intensive versus moderate lipid-lowering therapy on epicardial adipose tissue in hyperlipidemic post-menopausal women: A substudy of the BELLES trial (Beyond Endorsed Lipid Lowering with EBT Scanning). J. Am. Coll. Cardiol..

[168] Abe M., Matsuda M., Kobayashi H., Miyata Y., Nakayama Y., Komuro R., Fukuhara A., Shimomura I. (2008). Effects of statins on adipose tissue inflammation: Their inhibitory effect on MyD88-independent IRF3/IFN-beta pathway in macrophages. Arterioscler. Thromb. Vasc. Biol..

[169] Busnelli M., Manzini S., Froio A., Vargiolu A., Cerrito M.G., Smolenski R.T., Giunti M., Cinti A., Zannoni A., Leone B.E. (2013). Diet induced mild hypercholesterolemia in pigs: Local and systemic inflammation, effects on vascular injury-rescue by high-dose statin treatment. PLoS ONE.

[170] Horiuchi Y., Hirayama S., Soda S., Seino U., Kon M., Ueno T., Idei M., Hanyu O., Tsuda T., Ohmura H. (2010). Statin therapy reduces inflammatory markers in hypercholesterolemic patients with high baseline levels. J. Atheroscler. Thromb..

[171] Bonnet J., McPherson R., Tedgui A., Simoneau D., Nozza A., Martineau P., Davignon J., CAP Investigators (2008). Comparative effects of 10-mg versus 80-mg Atorvastatin on high-sensitivity C-reactive protein in patients with stable coronary artery disease: Results of the CAP (Comparative Atorvastatin Pleiotropic effects) study. Clin. Ther..

[172] González N., Moreno-Villegas Z., González Bris A., Egido J., Lorenzo Ó. (2017). Regulation of visceral and epicardial adipose tissue for preventing car diovascular injuries associated to obesity and dia betes. Cardiovasc. Diabetol..

[173] Wu C.K., Yeh C.F., Chiang J.Y., Lin T.T., Wu Y.F., Chiang C.K., Kao T.W., Hung K.Y., Huang J.W. (2017). Effects of atorvastatin treatment on left ventricular diastolic function in peritoneal dialysis patients-the ALEVENT clinical trial. J. Clin. Lipidol..

[174] Beck A.L., Otto M.E., D’Avila L.B., Netto F.M., Armendaris M.K., Sposito A.C. (2012). Diastolic function parameters are improved by the addition of sim vastatin to enalapril-based treatment in hyper tensive individuals. Atherosclerosis.

[175] Li J.J., Zheng X., Li J. (2007). Statins may be beneficial for patients with slow coronary flow syndrome due to its anti-inflammatory property. Med. Hypotheses.

[176] Choi S.Y., Park J.S., Roh M.S., Kim C.R., Kim M.H., Serebruany V. (2017). Inhibition of angiotensin II-Induced cardiac fibrosis by atorvastatin in adiponectin knockout mice. Lipids.

[177] Chen M., Li H., Wang G., Shen X., Zhao S., Su W. (2016). Atorvastatin prevents advanced glycation end products (AGEs)-induced cardiac fibrosis via activating peroxisome proliferator-activated receptor gamma (PPAR-g). Metabolism.

[178] Akahori H., Tsujino T., Naito Y., Matsumoto M., Sasaki N., Iwasaku T., Eguchi A., Sawada H., Hirotani S., Masuyama T. (2014). Atorvastatin ameliorates cardiac fibrosis and improves left ventricular diastolic function in hypertensive diastolic heart failure model rats. J. Hypertens..

[179] Yamamoto C., Fukuda N., Jumabay M., Saito K., Matsumoto T., Ueno T., Soma M., Matsumoto K., Shimosawa T. (2011). Protective effects of statin on cardiac fibrosis and apoptosis in adrenomedullin-knockout mice treated with angiotensin II and high salt loading. Hypertens. Res..

[180] Chen Y., Ohmori K., Mizukawa M., Yoshida J., Zeng Y., Zhang L., Shinomiya K., Kosaka H., Kohno M. (2007). Differential impact of atorvastatin vs pravastatin on progressive insulin resistance and left ventricular diastolic dysfunction in a rat model of type II diabetes. Circ. J..

[181] Yang X., Xiong T., Ning D., Wang T., Zhong H., Tang S., Mao Y., Zhu G., Wang D. (2020). Long-term atorvastatin or the combination of atorvastatin and nicotinamide ameliorate insulin resistance and left ventricular diastolic dysfunction in a murine model of obesity. Toxicol. Appl. Pharmacol..

[182] Werida R., Khairat I., Khedr N.F. (2021). Effect of atorvastatin versus rosuvastatin on inflammatory biomarkers and LV function in type 2 diabetic patients with dyslipidemia. Biomed. Pharmacother..

[183] Qie L., Meng X., Wang Y., Feng M., Zhong M., Li L. (2008). Assessment of regional systolic and diastolic functions affected by atorvastatin in coronary artery disease using tissue Doppler imaging. Clin. Cardiol..

[184] Preiss D., Campbell R.T., Murray H.M., Ford I., Packard C.J., Sattar N., Rahimi K., Colhoun H.M., Waters D.D., LaRosa J.C. (2015). The effect of statin therapy on heart failure events: A collaborative meta-analysis of unpublished data from major randomized trials. Eur. Heart J..

[185] Fukuta H., Goto T., Wakami K., Ohte N. (2016). The effect of statins on mortality in heart failure with preserved ejection fraction: A meta-analysis of propensity score analyses. Int. J. Cardiol..

[186] Liu G., Zheng X.X., Xu Y.L., Ru J., Hui R.T., Huang X.H. (2014). Meta-analysis of the effect of statins on mortality in patients with preserved ejection fraction. Am. J. Cardiol..

[187] Nochioka K., Sakata Y., Miyata S., Miura M., Takada T., Tadaki S., Ushigome R., Yamauchi T., Takahashi J., Shimokawa H. (2015). CHART-2 Investigators. Prognostic impact of statin use in patients with heart failure and preserved ejection fraction. Circ. J..

[188] Ashton E., Windebank E., Skiba M., Reid C., Schneider H., Rosenfeldt F., Tonkin A., Krum H. (2011). Why did high-dose rosuvastatin not improve cardiac remodeling in chronic heart failure? Mechanistic insights from the UNIVERSE study. Int. J. Cardiol..

[189] Kjekshus J., Apetrei E., Barrios V., Böhm M., Cleland J.G., Cornel J.H., Dunselman P., Fonseca C., Goudev A., Grande P. (2007). Rosuvastatin in older patients with systolic heart failure. N. Engl. J. Med..

[190] Jamialahmadi T., Simental-Mendia L.E., Eid A.H., Almahmeed W., Salehabadi S., Al-Rasadi K., Banach M., Sahebkar A. (2024). Efficacy of statin therapy in reducing epicardial adipose tissue: A systematic review and meta-analysis. Arch. Med. Sci..

[191] German C.A., Liao J.K. (2023). Understanding the molecular mechanisms of statin pleiotropic effects. Arch. Toxicol..

[192] Parisi V., Petraglia L., D’Esposito V., Cabaro S., Rengo G., Caruso A., Grimaldi M.G., Baldascino F., De Bellis A., Vitale D. (2019). Statin therapy modulates thickness and inflammatory profile of human epicardial adipose tissue. Int. J. Cardiol..

[193] Stary H.C., Blankenhorn D.H., Chandler A.B., Glagov S., Insull W., Richardson M., Rosenfeld M.E., Schaffer S.A., Schwartz C.J., Wagner W.D. (1992). A definition of the intima of human arteries and of its atherosclerosis-prone regions. A report from the Committee on Vascular Lesions of the Council on Arteriosclerosis, American Heart Association. Arterioscler. Thromb. Vasc. Biol..

[194] Ran C.Q., He W.T. (2025). Epicardial Adipose Tissue: A Potential Target to Improve Left Ventricular Diastolic Dysfunction. Rev. Cardiovasc. Med..

[195] Dozio E., Ruscica M., Vianello E., Macchi C., Sitzia C., Schmitz G., Tacchini L., Corsi Romanelli M.M. (2020). PCSK9 Expression in Epicardial Adipose Tissue: Molecular Association with Local Tissue Inflammation. Mediat. Inflamm..

[196] Rivas Galvez R.E., Morales Portano J.D., Trujillo Cortes R., Gomez Alvarez E.B., Sanchez Cubias S.M., Zelaya S.M. (2020). Reduction of epicardial adipose tissue thickness with PCSK9 inhibitors. Eur. Heart J..

[197] Malavazos A.E., Ermetici F., Cereda E., Coman C., Locati M., Morricone L., Corsi M.M., Ambrosi B. (2008). Epicardial fat thickness: Relationship with plasma visfatin and plasminogen activator inhibitor-1 levels in visceral obesity. Nutr. Metab. Cardiovasc. Dis..

[198] Maheswari G., Yamini B., Guru A., Velayutham M., Dhandapani V.E., Karuppiah K., Amalorpavanaden N.D. (2023). Trimetazidine with an adjuvant therapy to normalize the circulating visfatin concentration: Future perspective and mechanistic strategies. J. King Saud. Univ.–Sci..

[199] Chrusciel P., Rysz J., Banach M. (2014). Defining the role of trimetazidine in the treatment of cardiovascular disorders: Some insights on its role in heart failure and peripheral artery disease. Drugs.

[200] Salazar J., Luzardo E., Mejías J.C., Rojas J., Ferreira A., Rivas-Ríos J.R., Bermúdez V. (2016). Epicardial Fat: Physiological, Pathological, and Therapeutic Implications. Cardiol. Res. Pract..

[201] Patel V.B., Basu R., Oudit G.Y. (2016). ACE2/Ang 1-7 axis: A critical regulator of epicardial adipose tissue inflammation and cardiac dysfunction in obesity. Adipocyte.

[202] Shah S.A., Echols J.T., Sun C., Wolf M.J., Epstein F.H. (2022). Accelerated fatty acid composition MRI of epicardial adipose tissue: Development and application to eplerenone treatment in a mouse model of obesity-induced coronary microvascular disease. Magn. Reson. Med..

[203] Guo C., Ricchiuti V., Lian B.Q., Yao T.M., Coutinho P., Romero J.R., Li J., Williams G.H., Adler G.K. (2008). Mineralocorticoid receptor blockade reverses obesity related changes in expression of adiponectin, peroxisome proliferator-activated receptor gamma, and proinflammatory adipokines. Circulation.

[204] Wada T., Ishikawa A., Watanabe E., Nakamura Y., Aruga Y., Hasegawa H., Onogi Y., Honda H., Nagai Y., Takatsu K. (2017). Eplerenone prevented obesity-induced inflammasome activation and glucose intolerance. J. Endocrinol..

[205] Olivier A., Pitt B., Girerd N., Lamiral Z., Machu J.L., McMurray J.J.V., Swedberg K., van Veldhuisen D.J., Collier T.J., Pocock S.J. (2017). Effect of eplerenone in patients with heart failure and reduced ejection fraction: Potential effect modification by abdominal obesity: Insight from the EMPHASIS-HF trial. Eur. J. Heart Fail..

[206] Mitchell C.S., Premaratna S.D., Bennett G., Lambrou M., Stahl L.A., Jois M., Barber E., Antoniadis C.P., Woods S.C., Cameron-Smith D. (2021). Inhibition of the Renin-Angiotensin System Reduces Gene Expression of Inflammatory Mediators in Adipose Tissue Independent of Energy Balance. Front. Endocrinol..

[207] Anand I., Claggett B., Liu J., Shah A.M., Rector T.S., Shah S.J., Desai A.S., O’meara E., Fleg J.L., Pfeffer M.A. (2017). Interaction Between Spironolactone and Natriuretic Peptides in Patients with Heart Failure and Preserved Ejection Fraction: From the TOPCAT Trial. JACC Heart Fail..

[208] Anand I.S., Rector T.S., Cleland J.G., Kuskowski M., McKelvie R.S., Persson H., McMurray J.J., Zile M.R., Komajda M., Massie B.M. (2011). Prognostic value of baseline plasma amino-terminal pro-brain natriuretic peptide and its interactions with irbesartan treatment effects in patients with heart failure and preserved ejection fraction: Findings from the I-PRESERVE trial. Circ. Heart Fail..

[209] Moro C., Klimcakova E., Lolmède K., Berlan M., Lafontan M., Stich V., Bouloumié A., Galitzky J., Arner P., Langin D. (2007). Atrial natriuretic peptide inhibits the production of adipokines and cytokines linked to inflammation and insulin resistance in human subcutaneous adipose tissue. Diabetologia.

[210] Bae C.R., Hino J., Hosoda H., Arai Y., Son C., Makino H., Tokudome T., Tomita T., Kimura T., Nojiri T. (2017). Over expression of C-type natriuretic peptide in endothelial cells protects against insulin resistance and inflammation during diet-induced obesity. Sci. Rep..

[211] Tamura N., Ogawa Y., Chusho H., Nakamura K., Nakao K., Suda M., Kasahara M., Hashimoto R., Katsuura G., Mukoyama M. (2000). Cardiac fibrosis in mice lacking brain natriuretic peptide. Proc. Natl. Acad. Sci. USA..

[212] Lanfear D.E., Chow S., Padhukasahasram B., Li J., Langholz D., Tang W.H., Williams L.K., Sabbah H.N. (2014). Genetic and nongenetic factors influencing pharmacokinetics of B-type natriuretic peptide. J. Card. Fail.

[213] Standeven K.F., Hess K., Carter A.M., Rice G.I., Cordell P.A., Balmforth A.J., Lu B., Scott D.J., Turner A.J., Hooper N.M. (2011). Neprilysin, obesity and the metabolic syndrome. Int. J. Obes..

[214] Bordicchia M., Ceresiani M., Pavani M., Minardi D., Polito M., Wabitsch M., Cannone V., Burnett J.C., Dessì-Fulgheri P., Sarzani R. (2016). Insulin/glucose induces natriuretic peptide clearance receptor in human adipocytes: A metabolic link with the cardiac natriuretic pathway. Am. J. Physiol. Regul. Integr. Comp. Physiol..

[215] Wu W., Shi F., Liu D., Ceddia R.P., Gaffin R., Wei W., Fang H., Lewandowski E.D., Collins S. (2017). Enhancing natriuretic peptide signaling in adipose tissue, but not in muscle, protects against diet-induced obesity and insulin resistance. Sci. Signal.

[216] Park H.J. (2022). Anti-inflammatory effects of colchicine on coronary artery disease. Cardiovasc. Prev. Pharmacother..

[217] Egami Y., Nishino M., Shutta R., Makino N., Taniike M., Nakamura D., Yoshimura T., Mori N., Tanaka A., Morita H. (2014). Correlation between Colchicine Effects on Early Atrial Fibrillation Recurrence after Ablation and Left Atrium Epicardial Adipose Tissue Volume. J. Am. Coll. Cardiol..

[218] Lakshamanan S., Abbas N., Narashim A., Condello M., Kinninger A., Krishnan S., Roy S., Budoff M.J. (2026). 26-A-20880-ACC The Effect of Colchicine on Epicardial Adipose Tissue by Serial CCTA: Insights from the Ekstrom Trial. J. Am. Coll. Cardiol..

